# Radiochemistry, Production Processes, Labeling Methods, and ImmunoPET Imaging Pharmaceuticals of Iodine-124

**DOI:** 10.3390/molecules26020414

**Published:** 2021-01-14

**Authors:** Krishan Kumar, Arijit Ghosh

**Affiliations:** Laboratory for Translational Research in Imaging Pharmaceuticals, The Wright Center of Innovation in Biomedical Imaging, Department of Radiology, The Ohio State University, Columbus, OH 43212, USA; Arijit.Ghosh@osumc.edu

**Keywords:** positron emission tomography, PET, target-specific biomolecules, immunoPET imaging pharmaceuticals, production processes, ^124^I-labeled monoclonal antibodies, cancer, radiolabeling, radiotracers

## Abstract

Target-specific biomolecules, monoclonal antibodies (mAb), proteins, and protein fragments are known to have high specificity and affinity for receptors associated with tumors and other pathological conditions. However, the large biomolecules have relatively intermediate to long circulation half-lives (>day) and tumor localization times. Combining superior target specificity of mAbs and high sensitivity and resolution of the PET (Positron Emission Tomography) imaging technique has created a paradigm-shifting imaging modality, ImmunoPET. In addition to metallic PET radionuclides, ^124^I is an attractive radionuclide for radiolabeling of mAbs as potential immunoPET imaging pharmaceuticals due to its physical properties (decay characteristics and half-life), easy and routine production by cyclotrons, and well-established methodologies for radioiodination. The objective of this report is to provide a comprehensive review of the physical properties of iodine and iodine radionuclides, production processes of ^124^I, various ^124^I-labeling methodologies for large biomolecules, mAbs, and the development of ^124^I-labeled immunoPET imaging pharmaceuticals for various cancer targets in preclinical and clinical environments. A summary of several production processes, including ^123^Te(d,n)^124^I, ^124^Te(d,2n)^124^I, ^121^Sb(α,n)^124^I, ^123^Sb(α,3n)^124^I, ^123^Sb(^3^He,2n)^124^I, ^nat^Sb(α, xn)^124^I, ^nat^Sb(^3^He,n)^124^I reactions, a detailed overview of the ^124^Te(p,n)^124^I reaction (including target selection, preparation, processing, and recovery of ^124^I), and a fully automated process that can be scaled up for GMP (Good Manufacturing Practices) production of large quantities of ^124^I is provided. Direct, using inorganic and organic oxidizing agents and enzyme catalysis, and indirect, using prosthetic groups, ^124^I-labeling techniques have been discussed. Significant research has been conducted, in more than the last two decades, in the development of ^124^I-labeled immunoPET imaging pharmaceuticals for target-specific cancer detection. Details of preclinical and clinical evaluations of the potential ^124^I-labeled immunoPET imaging pharmaceuticals are described here.

## 1. Introduction

Several non-invasive imaging techniques are being used to identify, characterize, and quantify in vivo anatomical changes and biological processes that occur at the cellular and molecular levels. Radioisotope-based Positron Emission Tomography (PET), and Single-Photon Emission Computed Tomography (SPECT) are very sensitive imaging techniques. However, PET is considered to be superior to SPECT due to the availability of higher sensitivity scanners and better quantification of regional tissue concentrations of radiolabeled imaging pharmaceuticals [1]. Sufficient acquisition speed of the PET imaging technique allows the determination of pharmacokinetics and biodistribution of imaging pharmaceuticals and produces three-dimensional images of the functional processes in the body. 

Various non-metallic (^11^C, ^13^N, ^15^O, ^18^F, and ^124^I, etc.) and metallic (^64^Cu, ^68^Ga, and ^89^Zr, etc.) radionuclides are used routinely for the preparation of PET imaging pharmaceuticals for preclinical and clinical environments [2]. Table 1 provides a summary of the physical characteristics and the production methods [2,3,4,5] for some PET radionuclides, produced by a generator and proton bombardment of solid, liquid, and gas targets, that are suitable for radiolabeling of small and large biomolecules and nanomaterials for the development of potential PET imaging pharmaceuticals. 

The clinical applications of PET imaging pharmaceuticals have increased tremendously over the past several years since the availability of the FDA (Food and Drug Administration) approved ^11^C-, ^18^F-, and ^68^Ga-labeled imaging pharmaceuticals, ([^11^C]Choline, [^11^C]Acetate, [^18^F]NaF, [^18^F]FDG, [^18^F]Florbetapir, [^18^F]Fluemetamol, [^18^F]Florbetaben, [^18^F]Fluciclovine, [^18^F]Flortaucipir [^18^F]Fluoroestradiol, [^68^Ga]DOTA-TATE (NETSPOT), [^68^Ga]DOTA-TOC), and [^68^Ga]PSMA-11, for various applications, including metabolism, neurology, and oncology, etc. Additional worldwide clinical trials with ^68^Ga-labeled PSMA target-specific ligands, PSMA-11 and PSMA-617, are ongoing for prostate cancer imaging [6] The majority of clinical applications involve [^18^F]FDG; however, its use for neurological, oncological, and cardiological applications has been limited [7]. Therefore, numerous radiolabeled biomolecules that can target receptors that are known to overexpress on certain tumors were discovered, developed, and tested in the past [8,9,10].

Target-specific biomolecules, known to have high specificity and affinity for receptors associated with tumors and other pathological conditions, include small biomolecules (e.g., folate), peptides, and larger biomolecules like monoclonal antibodies (mAb), proteins, antibody fragments, and RNA nanoparticles [11,12,13]. The large biomolecules (mAbs and proteins etc.), with higher tumor specificity and affinity, have relatively intermediate to long circulation half-lives (>day) and tumor localization times. Combining superior target specificity of mAbs and high sensitivity and resolution of the PET technique has created a paradigm-shifting imaging modality, ImmunoPET (Immuno Positron Emission Tomography) [14]. The concept of immunoPET was proposed more than two decades ago. Significant progress has been made, since then, in the development of immunoPET imaging pharmaceuticals as a result of FDA approval of several therapeutics mAbs in recent years [15,16,17]. Our understanding of tumor heterogeneity and clinical disease management has improved, in the recent past, due to the availability of immunoPET. 

The critical factors that need to be considered for the selection of positron-emitting radionuclides for the development of immunoPET imaging pharmaceuticals are: (1) desirable decay characteristics of the radionuclide to yield high-quality images, (2) availability of methods to produce the isotope in sufficient and pure amounts, (3) availability of efficient radiolabeling methodologies, and most importantly, (4) physical half-life of the radionuclide that will allow sufficient time to monitor pharmacokintetics (tumor uptake and elimination) and biodistribution and for the transportation of the radiolabeled material to the preclinical or clinical site.

Short-lived and long-lived radioisotopes are considered suitable for the development of small-molecule- and large-biomolecule-based PET imaging pharmaceuticals, respectively, by using indirect and direct labeling techniques. Various strategies, including bifunctional chelating agents, prosthetic groups, click chemistry, enzyme-mediated, silicon- and boron- acceptor methodologies, the pre-targeting, the reporting gene methods, etc., are used for the design and development of immunoPET imaging pharmaceuticals [2,14]. The radionuclides with a short half-life (e.g., ^68^Ga and ^18^F) are unsuitable for the development of immunoPET imaging pharmaceuticals. Consequently, radionuclides with longer half-lives, with well-established radiolabeling methodologies, are used for the development of large biomolecules based imaging pharmaceuticals matching more closely with their longer circulation times. Some of the suitable metallic (e.g., ^44^Sc, ^52^Mn, ^55^Co, ^64^Cu, ^66^Ga, ^86^Y, ^89^Zr, etc.) and non-metallic (e.g., ^124^I) radionuclides with longer half-lives are listed in Table 1.

Thermodynamically stable and kinetically inert radiolabeled metal (e.g., ^64^Cu, ^89^Zr, ^66^Ga, and ^86^Y, etc.) chelate conjugates, using bifunctional chelating agents (BFC) to target-specific biomolecules, have been studied extensively for their potential applications as imaging pharmaceuticals. Two steps are involved in the development of metallic radionuclide-labeled large-biomolecules based imaging pharmaceuticals. The first step is the conjugation of a bifunctional chelating agent that forms a thermodynamically stable and kinetically inert metal chelate, with the target-specific large biomolecule. In the second step, the BFC-large biomolecule conjugate is labeled with a metallic radionuclide [18,19,20]. Linear (e.g., DTPA = Diethylenetriamine N, N, N′, N″, N‴ pentaacetic acid, HBED-CC = N,N′-bis[2-hydroxy-5-(carboxyethyl)benzyl] ethylenediamine-N,N′-diacetic acid, and DFO = Desferrioxamine B or Deferoxamine B, etc.) and macrocyclic polyaminocarboxylates (e.g., NOTA = 1,4,7-triazacyclononane-1,4,7-triacetic acid, DOTA = 1,4,7,10-tetraazacyclododecane-1,4,7,10-tetraacetic acid, etc.) and their analogs and derivatives are known to form thermodynamically stable and kinetically inert metal chelates. Alternatively, the radiolabeling of the BFC is accomplished in the first step followed by the conjugation to the target-specific biomolecule in the second step.

Based on the long half-life and physical properties of the positron-emitting isotope of iodine, ^124^I may be used for both imaging (positron) and therapy (electron capture) as well as for ^131^I dosimetry. The therapeutic effect of ^124^I relies on the Auger electron emission responsible for the local action within nanometers. The relatively low percentage of high-energy positrons (22.7%) and a high percentage of cascade gamma photons in the background compared to the conventional PET isotopes makes imaging with ^124^I technically challenging. However, optimizing image acquisition parameters and appropriate corrections within the image reconstruction process improve the image quality. ^89^Zr and ^52^Mn, with 3.27 and 5.59 d half-lives, respectively, are attractive choices for the development of immunoPET imaging pharmaceuticals. Labeling of large biomolecules with metallic radionuclides requires additional conjugation steps in the process and purification could be challenging. DFO has been the most popular bifunctional chelator for conjugation with mAbs and ^89^Zr labeling. The production of high purity ^52^Mn and in vivo stability of manganese chelates and their conjugates are still developing.

I-124 is an attractive radionuclide for the development of mAbs as potential immunoPET imaging pharmaceuticals due to its physical properties (decay characteristics and half-life), easy and routine production by cyclotrons [21], and well-established methodologies for radioiodination [22,23,24]. For example, ^124^I has been used to label small molecules, peptides, mAbs, proteins, and antibody fragments for tumor imaging [25,26,27,28], in thyroid and parathyroid cancer imaging [29,30,31], to label single molecules like meta-iodobenzylguanidine (MIBG), amino acids, and fatty acids among others for investigation of several heart and brain diseases, as well as functional studies of neurotransmitter receptors [32,33,34]. It has also been used to label photosensitizers for photodynamic therapy [35]. Labeling of biomolecules with radioactive iodine was first established several decades ago when the ^131^I isotope of iodide was used for labeling polyclonal antikidney serum [36]. The aromatic moieties present in the large biomolecule to be labeled are a tyrosine residue and to a lesser extent a histidyl group [37,38].

The objective of the present report is to provide a comprehensive review of the physical properties of iodine and ^124^I radionuclide, production processes of ^124^I radionuclide, various ^124^I-labeling methodologies for large biomolecules, specifically mAbs, and application of ^124^I-labeled mAb, as immunoPET imaging pharmaceuticals, for oncologic applications. A summary of several production processes, including ^123^Te(d,n)^124^I, ^124^Te(d,2n)^124^I, ^nat^Sb(α,xn)^124^I, ^121^Sb(α,n), natSb(^3^He,n) reactions, a detailed overview of the ^124^Te(p,n)^124^I reaction (including target selection, preparation, processing, and recovery of ^124^I), and a fully automated process that can be scaled up for GMPproduction of large quantities of ^124^I is provided. Direct, using inorganic and organic oxidizing agents and enzyme catalysis, and indirect, using prosthetic groups, ^124^I-labeling techniques, have been discussed. Significant research has been conducted, in more than the last two decades, in the development of ^124^I-labeled immunoPET imaging pharmaceuticals for target-specific cancer detection. Details of preclinical and clinical evaluations of the potential ^124^I-labeled immunoPET imaging pharmaceuticals are described here.

Overall, this is a first comprehensive review providing a thorough understanding of various areas that are essential for our understanding of the discovery and the development of novel ^124^I-labeled immunoPET imaging pharmaceuticals.

## 2. Overview of Physical Properties of Iodine and Iodine Radionuclides

Iodine with symbol I, atomic number 53, and atomic weight 127, and with [Kr]4d^10^5s^2^5p^5^ electronic configuration belongs to group 17 of the periodic table. Iodine exists as a diatomic molecule, I_2_, in its elemental state and is known to exist in −1, +1, +3, 5, and 7 oxidation states. Atomic radii are 133 and 220 pm for Iodine (I_2_) and iodide (I^−^), respectively. Elemental iodine, with chemical formula I_2_, where two iodine atoms share a pair of electrons to each achieve a stable octet for themselves. Similarly, the iodide anion, I^−^, is the strongest reducing agent among the stable halogens, being the most easily oxidized back to diatomic I_2_. In general, I_2_ converts into I_3_^−^ in the presence of excess iodide. The standard potential of the iodide/triiodide redox couple is 0.35 V (versus the normal hydrogen electrode, NHE) [39].

Iodine radioisotopes have long been used as theranostic agents in the field of thyroid cancer [40]. There are 37 known isotopes of iodine, ^108^I to ^144^I, that undergo radioactive decay, except ^127^I which is a stable isotope. The longest-lived of the radioactive isotopes of iodine is ^129^I with a 15.7 million years half-life, decaying via beta decay to stable ^129^Xe [41]. However, the most well-known iodine radionuclides are ^123^I, ^124^I, ^125^I, and ^131^I, which are used in preclinical and clinical environments for medical applications. Background related to their physical characteristics and medical applications are given below.

The main gamma emission peak of ^123^I, 159 keV, makes it suitable for SPECT imaging as it is close to the 140 keV peak of ^99m^Tc peak. A short physical half-life (13.22 h) [41] of ^123^I allows the study of compounds that have rapid radiolabeling methods, fast clearance, and short metabolic processes. Several ^123^I-labeled imaging pharmaceuticals, including ^123^I-Iobenguane, for detection of primary or metastatic pheochromocytoma or neuroblastoma as an adjunct to other diagnostic tests, ^123^I-ioflupane for visualization of the striatal dopamine transporter, and [^123^I]NaI capsules for evaluation of thyroid function and morphology, are approved by the Food and Drug Administration (FDA) for clinical use.

I-125 has mainly X-ray energy emission at 27 keV with low gamma emission at 35.5 keV. It has photon energy which is low for optimal imaging and its half-life is long (59.4 days) [41]. ^125^I-labeled imaging pharmaceuticals, ^125^I-HSA and ^125^I-iothalamate, are approved by the FDA for clinical use for total blood/plasma determination and evaluation of glomerular filtration, respectively. This radionuclide is used routinely in discovery and preclinical environments. For example, ^125^I has been used for NCEs (New Chemical Entities) labeling and their evaluation for in vitro cell binding assays, biodistribution, and pharmacokinetic properties in preclinical models.

I-131, a beta-emitting isotope (606 keV, 90%) and a half-life of 8.02 days [41], is often used for radiotherapy. The penetration range of the beta particle is 0.6 to 2.0 mm at the site of uptake. The ^131^I is taken up into thyroid tissue. The beta particles emitted by the radioisotope destroy the associated thyroid tissue with little damage to surrounding tissues (more than 2.0 mm from the tissues absorbing the iodine). ^131^I emits gamma photons that can be used for SPECT imaging. ^131^Iodine meta-iodobenzylguanidine (^131^I-MIBG) is a radiopharmaceutical used for both imaging and treating certain types of neuroendocrine tumors, including neuroblastomas, paragangliomas, and pheochromocytomas. FDA approved ^131^I-labeled products are, iobenguane ^131^I, a form of ^131^I-MIBG, for the treatment of paragangliomas and pheochromocytomas, ^131^I-labeled HSA for determination of total blood and plasma volumes, cardiac output, cardiac and pulmonary blood volumes and circulation times, protein turnover studies, heart and great vessel delineation, localization of the placenta, and localization of cerebral neoplasms, and [^131^I]NaI for the diagnostics and the therapeutic applications.

Initially, ^124^I was considered as an impurity in the production of ^123^I, although it was recognized that this radionuclide has attractive properties for use in PET imaging. For example, the half-life of 4.18 d is long enough for clearance and localization of ^124^I-labeled mAbs. Additionally, the 22.7% positron decay with maximum and mean positron energies of 2.138 and 0.975 MeV, respectively, allows PET imaging. In contrast, the most common PET radiotracer, ^18^F, has a positron abundance of 97% with maximum and mean positron energies of 0.634 and 0.250 MeV, respectively. ^1^^24^I has potential as both diagnostics and therapeutic radionuclide and its use are becoming more widespread.

**Figure 1:**
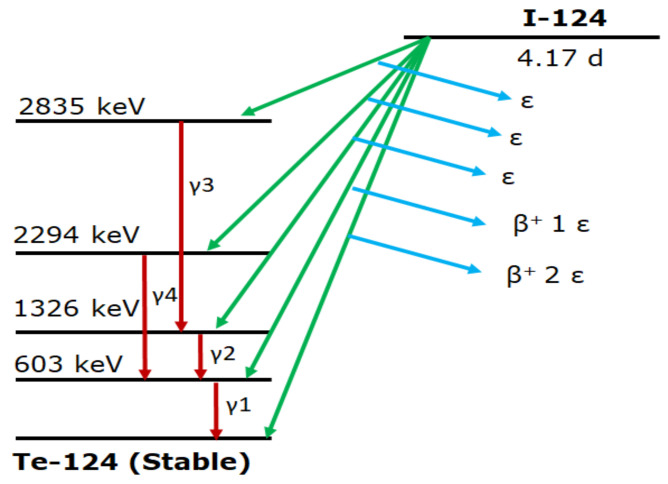
Simplified decay scheme of ^124^I radionuclide (taken from reference [42]).

In addition to positron emissions, ^124^I emits a rather large portion of gamma rays during its decay (Figure 1), with the majority (63%) of which is 603 keV energy (Table 2). Coincidences of this 603 keV photon and a 511 keV annihilation photon cannot be distinguished from the true coincidences involving two 511 keV annihilation photons. Multiple correction methods have been suggested to address this background activity but their effectiveness is limited in the setting of the low count rates observed in clinical scans.

## 3. Overview of ^124^I Production Processes

Routine availability of a long half-life radioisotope (^124^I) for PET imaging that is economically, efficiently, and safely produced will enable the evaluation and development of numerous immunoPET imaging pharmaceuticals for research and clinical use. The planned strategies for the production of ^124^I at a particular facility are decided by the availability of irradiating particles and their energy ranges. If multiple choices of beams are available at the production site, a reaction scheme is selected which produces ^124^I with maximum yield and highest purity.

### 3.1. Production Reactions, Target Selection, and Preparation

Early investigations were focused on the production, including excitation functions determinations, of ^124^I from the deuterium, α, ^3^He irradiation of Te and Sb solid targets, including ^123^Te(d,n)^124^I, ^124^Te(d,2n)^124^I, ^121^Sb(α,n)^124^I, ^123^Sb(α,3n)^124^I, ^123^Sb(^3^He,2n)^124^I, ^nat^Sb(α, xn)^124^I, ^nat^Sb(^3^He,n)^124^I reactions [22,43,44,45,46,47,48,49,50,51,52,53,54,55,56,57,58,59,60,61,62]. Detailed background related to these production processes is reported in two excellent reviews [22,43]. As a result of less frequent availability of deuteron, alpha, and ^3^He beams and high ^125^I content in the produced materials by these reactions, these are not routinely used for ^124^I production in the research and clinical environments. Significant interest grew in the ^124^Te(p,n)^124^I reaction, despite a slightly lower yield than the ^124^Te(d,2n)^124^I reaction, after a careful study of the process involving cross-section measurements and the production experiments [63,64,65,66,67,68,69]. The first ^124^I production process was proposed based on the ^123^I production method which involved 3–8 h irradiation of a ^124^Te containing capsule, and irradiated by a ~26 MeV 8–18 µA proton beam. A similar capsule target was irradiated with a 12 MeV proton beam for the production of ^124^I. The target was processed chemically to isolate ^123/124^I [70].

Numerous studies were conducted since the first report by Kondo et al. in 1977 [70]. The results from these studies suggested that ^124^I can be successfully produced for research and clinical use by using low-energy cyclotrons that are available routinely for production of ^11^C and ^18^F-labeled imaging pharmaceuticals for standard care [71,72,73,74,75,76,77,78,79,80,81,82,83,84,85,86]. Consequently, the ^124^Te(p,n)^124^I production process is being used extensively in the research and clinical environments worldwide. The focus of this report will be to review the progress and the status of the ^124^I production by using the ^124^Te(p,n)^124^I reaction. Other potential reactions that have been proposed and considered for the production of ^124^I are ^125^Te(p,2n)^124^I and ^126^Te(p,3n)^124^I. [61,87,88,89,90,91] A summary of some of the recent studies is provided in Table 3.

The main goal of any production process is to ensure that it produces the final product with the highest purity and yield. Consequently, it is important to fully understand the background related to the enrichment and purity requirements of the tellurium target and the production process parameters, i.e., irradiation energy and current of the proton beam, and target processing and recovery. The percent of natural abundance of various isotopes (given in the parenthesis) of ^nat^Te has been reported as ^120^Te (0.09%), ^122^Te (2.55%), ^123^Te (0.89%), ^124^Te (4.74%), ^125^Te (7.07%), ^126^Te (18.84%), ^128^Te (31.74%), and ^130^Te (34.08%) [69]. Irradiation of a ^nat^Te target with proton beam will, consequently, produce a mixture of various unwanted iodine isotopes with long half-lives, making the production and purification process inefficient and challenging and the ^124^I produced being unusable. Many reports exist on the proton-induced reactions on ^nat^Te. These reports are valuable for testing nuclear model calculations, integral data validation and some other applications, but not for routine production of high purity ^124^I for medical applications [91,92,93,94,95,96].

Therefore, a highly enriched ^124^Te target (>99% or better) material must be used for the production of ^124^I to minimize unwanted iodine isotopic impurities; although the cost of enriched tellurium increases significantly with the increased enrichment imposing the need for recycling the irradiated target material. In our laboratories, we have used the enriched target material with the following specifications: ^124^Te (99.3%), ^120^Te, ^122^Te, ^126^Te (<0.01%), ^123^Te (<0.05), ^128^Te (0.03%), ^130^Te (0.02), and ^125^Te (0.6%). Since the major contaminant in the enriched target is ^125^Te, one should investigate the production of potential radionuclides from ^125^Te(p,n)^125^I, ^125^Te(p,2n)^124^I, and ^125^Te(p,3n)^123^I reactions also.

The tellurium target is available either as metallic tellurium or TeO_2_. TeO_2_ is used routinely for ^124^I production due to better thermal characteristics than tellurium metal and to avoid evaporation of radioiodine. The melting points of TeO_2_ and Te are 733 and 449.5 °C, respectively [74,82]. Additionally, tellurium tends to blow up upon heating. The tellurium target for proton irradiation has been prepared by the three different methods: (1) filling ^124^Te in an aluminum capsule under He atmosphere [70,84], (2) introducing melted enriched tellurium onto a support plate [71,72,73,74,75,76,77,78,79,80,81,82,86], and (3) electroplating tellurium on a nickel-coated copper substrate [45,48,97,98].

In the second method of target preparation, the isotopically enriched tellurium ismelted onto a small platinum plate. The platinum surface should not be smooth rather be scratched with a scalpel or lancet before preparation of the target. An optimized amount of tellurium is critical for the quality of the target. Powdered Al_2_O_3_ (5–7%) is commonly mixed with TeO_2_ [74,77,79,80,81,82,86] during target preparation by melting for (1) increasing the heat transfer characteristics, (2) enhancing the TeO_2_ binding to the target plate [74,77,79], (3) giving the target material a glassy solid structure and eliminating the need for a cover foil [53,59], and (4) increasing the uniformity of the target material layer. Several binary tellurium compounds, to improve the thermal properties, were used, in the past, for the development of ^124^I production processes, including Al_2_Te_3_ [82] and Cu_2_Te [73,75] with 895 and 1132 °C melting points, respectively. Al_2_Te_3_ appeared to be a promising target material, providing a high tellurium mass fraction and a glassy melt material [82].

Higher beam currents can be used for the bombardment of the target when the tellurium is electroplated on a suitably large area of the target carrier and when a small beam/target angle irradiation is performed under the optimum cooling conditions. Large area electroplated tellurium targets are attractive for this application as long as the deposits are smooth, homogeneous, and free of other constituents. A new plating technology involving CCE (Constant Current Electrolysis) was developed to avoid the poor quality target layers during plating procedures [97]. In this method, tellurium targets were prepared by DC-CCE of the metal from alkaline plating solutions. 50 µm nickel-coated, needed for good adhesion of the target material, copper plates were used for target preparation. Details of this technique are presented elsewhere [97]. A mean weight of 90 ± 9 mg of enriched tellurium was deposited per target. The electroplating process is more expensive and requires more work and a precise set-up, but it may produce higher yields for the production of ^124^I. On the other hand, the melting process is experimentally simpler and produces targets that can be reused several times.

In addition to the selection of the target material and method of target preparation, various support plates, in which the target material is deposited either by melting or electrodeposition, have been used. These include Aluminum [45], Platinum [71,74,77,78,80,99], tantalum and nickel electroplated tantalum [100], nickel electroplated copper [44,47,98], tungsten and silicon [73], platinum-coated tungsten [75], platinum/iridium [47,49,50,72], and rhodium electroplated stainless steel [101]. Nickel-coated, to ensure good adhesion of tellurium, copper is a good target material for electrodeposition of tellurium to provide a good cooling efficiency during irradiation. This is due to its high melting point (1084.62 °C) and the high thermal conductivity (401 W m^−1^ K^−1^) of copper; although there are some disadvantages of using copper plating. Natural copper consists of ^65^Cu(30.83%) and ^63^Cu(69.17%), which have potential to produce different zinc isotopes from ^65^Cu(p,n)^65^Zn, ^65^Cu(p,2n)^64^Zn, ^63^Cu(p,n)^63^Zn, and ^63^Cu(p,2n)^62^Zn with ^65^Zn being long-lived (half-life = 244 d). The maximum cross-section for the ^65^Cu(p, n)^65^Zn reaction is around 11 MeV, which is in the same energy range as for the ^124^Te(p,n)^124^I reaction. Depending on the target thickness, the cross-section for the ^65^Cu(p,n)^65^Zn will be high enough to produce ^65^Zn. Consequently, a careful target design is required while using the nickel electroplated copper backing for tellurium electroplating. Platinum is considered a better choice as a coating or backing material for target preparation due to the fact that (1) it is not dissolved during the chemical processing to recover ^124^Te, (2) it is not necessary to make one target per irradiation, (3) the recovered ^124^Te may have a higher chemical purity, (4) it has a high melting point (1768 °C), which makes it suitable for a dry distillation of iodine. But it also has some disadvantages: it is more expensive than Cu and it has a much lower thermal conductivity (71.6 W·m^−1^·K^−1^) than copper.

The target thickness optimization and its orientation are two critical parameters during irradiation of the target for ^124^I production for high yield and purity. The optimized thickness is important to (1) ensure that the entire beam energy is not deposited within the target itself, (2) reduce the production of unwanted radioiodine impurities, and (3) reduce the cost of production. Additionally, the orientation of the target is also optimized to reduce the power density, which increases both the area over which the heat is deposited and the effective target thickness.

### 3.2. Proton Beam Energy and Current for Target Irradiation 

**Figure 2:**
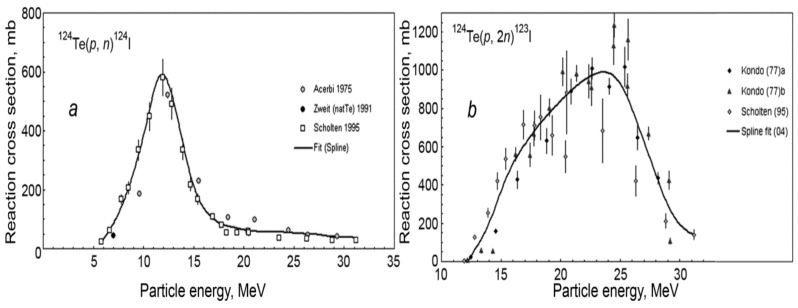
Comparison of reaction cross sections for the ^124^Te(p,n)^124^I and ^124^Te(p,2n)^123^I reactions (taken from reference [21]).

Proton irradiation parameters, i.e., proton beam energy, current, and irradiation time, are important parameters in maximizing the yield and minimizing the number and amount of impurities even if the 100% enriched ^124^Te target material is used. A recent study reported the calculation of the excitation functions for production of ^123^I and ^124^I from proton bombardment of ^124^Te by using TALYS 1.6[67] and comparing with the experimental results reported previously [64,70]. The calculated ^124^Te(p,n)^124^I reaction cross sections were in good agreement with the experimental data with a peak at ~600 mb [64]. The production of ^124^I is appropriate for small, medium-sized cyclotrons. Figure 2 shows a comparison of cross-section data for ^124^Te(p,n)^124^I and ^124^Te(p,2n)^123^I reactions [21]. The calculated cross-section data for ^124^Te(p,2n)^123^I reaction, shown in Figure 2, are in fairly good agreement with experimental data with a peak over 900 mb. However, there are some discrepancies in low and high energy regions (10–18 MeV, 25–30 MeV). Since there is an overlap between ^124^Te(p,n)^124^I and ^124^Te(p,2n)^123^I cross-section curves in the 12 to 16 MeV energy range; therefore, the proton bombardment of ^124^Te always produces a mixture of ^124^I and ^123^I. Since the decay of ^123^I is 7.6 times faster than ^124^I, overnight storage of the mixture is required in the production process for removal of ^123^I improving the purity of ^124^I; although it decreases the overall yield of ^124^I production.

**Figure 3:**
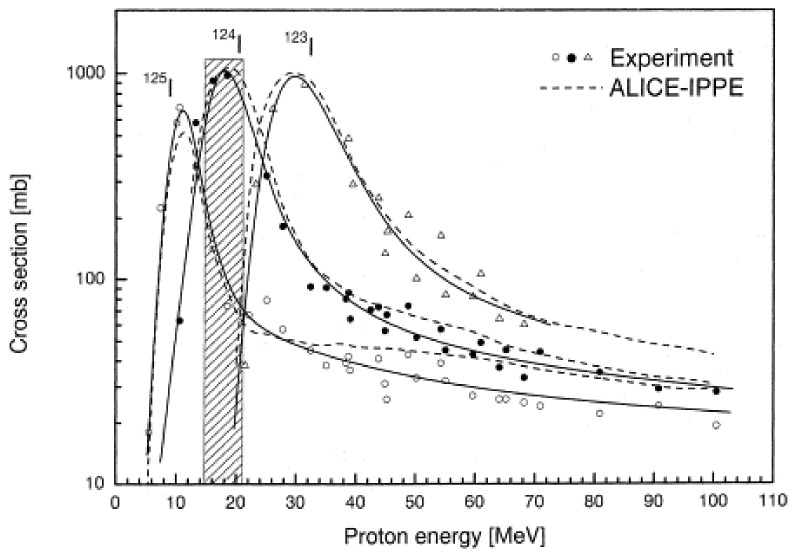
Excitation functions of ^125^Te(p,xn)^123,124,125^I reactions (taken from reference [65]). The broken lines show the results of nuclear model calculations using the code ALICE-IPPE. The shaded area gives a suitable energy range for the production of ^124^I.

Excitation functions of the ^125^Te(p,xn)^123,124,125^I nuclear reactions were measured, using targets that were prepared by electrolytic deposition of 98.3% enriched ^125^Te on a Ti-backing, in the threshold to 100 MeV energy range by using the stacked-foil techniques [65]. Additionally, the excitation functions were calculated by a modified hybrid model code ALICE-IPP. Figure 3 shows a plot of cross-section vs. incident proton energy for ^125^Te(p,n)^125^I, ^125^Te(p,2n)^124^I, and ^125^Te(p,3n)^123^I reactions. The experimental and calculated data agreed with each other. The data given in Figure 3 and integral yield data suggested that ^124^I and ^125^I are produced by low energy proton irradiation (<20 MeV). ^123^I is produced at >20 MeV. The energy 21→15 MeV appears to be suitable for ^124^I production from the ^125^Te(p,2n)^124^I reaction which is above the range of low energy cyclotrons. Below 15 MeV, the yield of ^124^I from ^125^Te(p,2n)^124^I reaction is low, and ^125^I from the ^125^Te(p,n)^125^I reaction is high. The ^123^I impurity is not a problem for ^125^Te(p,2n)^124^I reaction as (1) ^125^Te(p,3n)^123^I reaction requires >20 MeV, and (2) it decays out rather fast. The formation of ^125^I impurity, from ^125^Te impurity, in the ^124^Te(p,n)^124^I nuclear reaction is more critical and must be controlled. It has been reported that the yield of ^125^Te(p,2n)^124^I reaction is four times higher than ^124^Te(p,n)^124^I reaction with some ^125^I present making it an attractive route for ^124^I production [65]. However, the proposed production energy range is too high for small cyclotrons requiring medium-sized commercial cyclotrons.

For the energy range window employed for proton irradiation of ^124^Te enriched target using low energy cyclotrons, the primary reactions to consider are: ^124^Te(p,n)^124^I, ^124^Te(p,2n)^123^I, ^125^Te(p,n)^125^I, and ^125^Te(p,2n)^124^I. Given the difference in the half-lives of ^123^I (13.2 h) and ^125^I (59.4 days), a mixture of ^123^I and ^124^I will produce high purity ^124^I upon storage of the crude product overnight. On the other hand, a mixture of ^124^I and ^125^I will give ^124^I with lower purity with time, as the half-life ^124^I decay is 15 times faster than ^125^I decay. Consequently, it is critical to select an optimum proton beam energy to maximize the yield and purity of ^124^I, i.e., the lowest amounts of ^123^I and ^125^I, as there is a possibility of competing reactions during proton irradiation.

The ^123^I contaminant arising from the ^124^Te(p,2n)^123^I reaction may be minimized by reducing the incident proton energy. A decrease in energy from 13 MeV to 11 MeV results in a nearly three-fold decrease in the ^124^I yield [81]. To minimize these impurities, the exit energy is controlled by varying the thickness of the target material or by degrading the incident proton beam energy using aluminum foils. For example, it is expected that the 16.5 MeV proton energy is degraded to 14.4, 13.1 MeV, and 12.0 MeV by using 320 μm, 500 μm, or 640 μm aluminum foils, respectively [102]. It is critical to use an optimum thickness of the aluminum foil to ensure the highest yield and purity of ^124^I produced. Lamparter and coworkers [86] demonstrated that the irradiation of the ^124^Te solid target with a 10–15 μA proton beam degraded by a 320 μ foil resulted in an unfavorable ^123^I/^124^I ratio of 0.6–0.9. Introduction of a 640 μ thick foil produced ^124^I with extremely high radionuclidic purity but with low yield. Using 500 μ foil and 10 and 12 μA beam current produced acceptable results, Under these conditions, up to 150 MBq (*n* = 12) of no-carrier added [^124^I]NaI was produced after a 2 h irradiation time [86].

In general, the radionuclide produced from the proton bombardment of the target is dependent on the current intensity of the beam. However, there are certain limitations as to how much maximum current can be used for irradiation of the target which is dependent on the target material and the properties of radionuclide produced. For example, ^124^I produced from the ^124^Te(p,n)^124^I reaction is directly proportional to the amount of current at which the target is irradiated. Various studies reported in the literature have used 8–29 µA beam current. Lamparter et al. [86]. reported a process for ^124^I production using 10 and 12 uA proton beam irradiation for 2 h. However, the maximum current at which the ^124^Te target can be irradiated is dictated by the thermal performance of the target material, i.e., in some cases melting of tellurium and the volatility of ^124^I have been observed [82,103,104].

Due to the thermal stability of the target and volatility of ^124^I, extensive and efficient cooling of the target material and the support plate is accomplished by using water for the back of the target and helium for the front of the target material [74,77,80]. Front water cooling has been also tried but was found unsuitable for a target system design where the target was perpendicular to the proton beam. Relatively high losses of ^124^I, during extended irradiation period, to the cooling water directly in contact with the target were observed [77]. Computer simulation studies have been conducted to model heat transport during target irradiation [103].

### 3.3. Target Processing and Recovery of ^124^I

A chemical separation technique was used for the recovery of ^123/124^I from the irradiated ^124^Te target initially [70]. However, separation of ^124^I from irradiated solid ^124^Te target, which is fabricated by melting method, is routinely accomplished by the dry distillation method. The method is straightforward and allows easy recycling of the target [50]. To ensure maximum recovery of the target material and extracting maximum ^124^I, wide variation in setup parameters for distillation were reported, i.e., distillation time [50,74,78] and temperatures [74,87] being between 5 to 20 min and 670 to 820 °C, respectively. Similarly, a variation of carrier gases (Air [47,77,80], Argon [79], Helium [101], and Oxygen [74,78,93]) and their flow rates (5–80 mL/min) [50,74,79,80,81,87] were also reported in the literature for optimization of the method. Glaser et al. [78]. preferred an oxygen atmosphere for converting any tellurium, due to reduction, to TeO_2_ for recovery of the target. Two types of traps have been used in the past that includes a 100–1000 µL solution of 0.001–0.1 N Sodium Hydroxide [77,80,87] or stainless steel [47,50], pyrex [74], or quartz [49,81] capillary tube coated with sodium hydroxide. To increase the surface area of the capillary tube, a platinum wire was loaded into it [55]. The adsorbed ^124^I inside the capillary tube was washed with a weak buffer solution [74,81].

The IAEA (International Atomic Energy Agency) technical reports described two procedures of extraction of ^124^I from the irradiated targets prepared by the two methods, melting technique and electroplating [97]. In the first procedure, the irradiated target was introduced into a quartz tube horizontally mounted in a cylindrical mini-furnace with carrier gas flow. The carrier gas flow and the power supply of both the furnace and the heating element around the narrow quartz tube were turned on. The iodine was vaporized at about 620 °C from the target and trapped downstream in a vial that contained 0.01 N NaOH. The distillation rate of ^124^I from the ^124^Te target was controlled by the diffusion of iodine from the target surface. Between 710 and 740 °C (MP TeO_2_, 733 °C), an iodine vapor releases from the target. Therefore, 10 min after the start of the distillation, the furnace power supply was switched on and off so that the temperature oscillates between 700 and 740 °C. Periodic melting and solidification of the target resulted in a 98% recovery of the radioiodine and losses of TeO_2_ were limited to less than 0.2% [97].

In the second method described in the IAEA report, [44,97] the irradiated electroplated target layer was dissolved in an oxidized alkaline medium containing NaOH, H_2_O_2_, and water followed by a reduction of an enriched target to metal by aluminum powder. After processing the mixture and removal of tellurium and aluminum hydroxide, the solution was filtered through a 0.45 µm glass filter and an in-line AG 50 WX8 cation-exchange (H^+^ form, 100–200 mesh grade, 1 cm × 5 cm) column. When more than 5% of the iodine activity remained on the column, the latter was washed with 5 mL Milli-Q water. The eluate was collected into a pre-weighted serum vial. The overall yield of the chemical processing was higher than 95%.

### 3.4. A Fully-Automated Production Process for ^124^I

**Figure 4:**
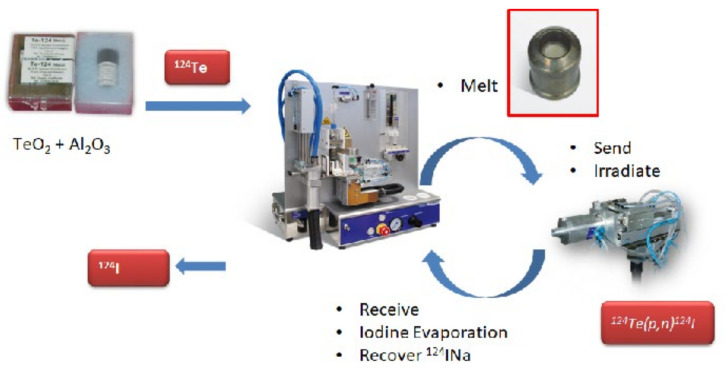
The schematic process diagram for the production of ^124^I from ^124^Te(p,n)^124^I reaction using Comecer ALCEO halogen system (Courtesy Comecer S.p.A.).

Tremendous progress has been made in the recent past in the development of a fully automated process. A fully-automated process, developed by Lamparter et al. [86], involves three different steps: (1) the preparation of the target in a shuttle, (2) the irradiation of the target, and (3) the processing of the irradiated target shuttle using a Comecer ALCEO Halogen system. The processing of the shuttle consists of two steps, (1) the extraction of ^124^I out of the target, and (2) elution of the trapped ^124^I into a product vessel. The Comecer ALCEO system consists of two different parts, the evaporation unit (EVP), which is used for the preparation of the target in the shuttle and processing of the irradiated shuttle, and the irradiation unit (PTS) with a supporting cooling unit. There is no intervention of an operator, during irradiation, target processing, and recovery of ^124^I [86]. A schematic process diagram is given in Figure 4.

**Figure 5:**
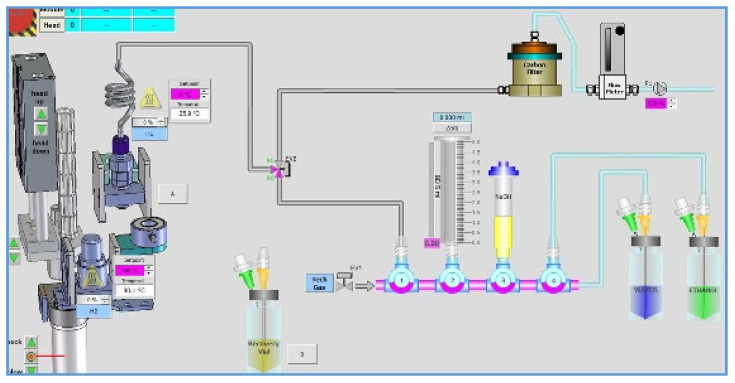
Recovery of ^124^I from irradiated ^124^Te target in a Comecer ALCEO halogen evaporation unit (EVP) module (courtesy of Comecer S.p. A.).

In the automated process reported by Lamparter et al. [86], a solid target was prepared by mixing 300 mg enriched [^124^Te]TeO_2_ (99.93%) and 15 mg neutral alumina powder (Al_2_O_3_). The target material, ^124^TeO_2_/Al_2_O_3_, was sintered into the shuttle as a 10 mm diameter circle with an estimated 4 mm^3^ size melt. For the irradiation, the shuttle was transferred, fully automatically via a tube system, to the irradiation unit PTS, connected to a 16.5 MeV GE PETtrace cyclotron, while undercooling. The shuttle was irradiated using a 10 and 12 μA current for 2 h. A 500 μ aluminum foil was used for an optimum thick target yield. The backside of the shuttle was cooled by water and the front was cooled by a constant Helium flow. After irradiation, the shuttle was transferred back to the ALCEO Halogen EVP module. ^124^I was extracted by heating the shuttle to 740 °C for 10 min and trapping of the vaporized ^124^I into a glass tube. The trapped ^124^I was eluted in the form of [^124^I]Iodide Sodium ([^124^I]NaI) with 500 μL aqueous 0.05 N NaOH. The whole procedure, including evaporation and extraction of ^124^I, was completed in 90 min. An extraction process of ^124^I is shown in Figure 5.

## 4. Overview of ^124^I-Labeling Methods

Numerous methods for radioiodination of small and large biomolecules, i.e., mAbs, have been reported in the past [105,106]. Regardless of the application, a radioimmunoassay reagent for in vitro testing or in vivo use as a diagnostic or therapeutic agent, greater care and testing is required to maintain immunoreactivity of the biomolecule followed by radiolabeling and purification. Achieving high molar activity of the radioiodinated biomolecule remains very important due to the necessity to target very low concentrations of specific targets and to avoid non-specific binding.

### 4.1. Direct Labeling Methods 

**Figure 6:**
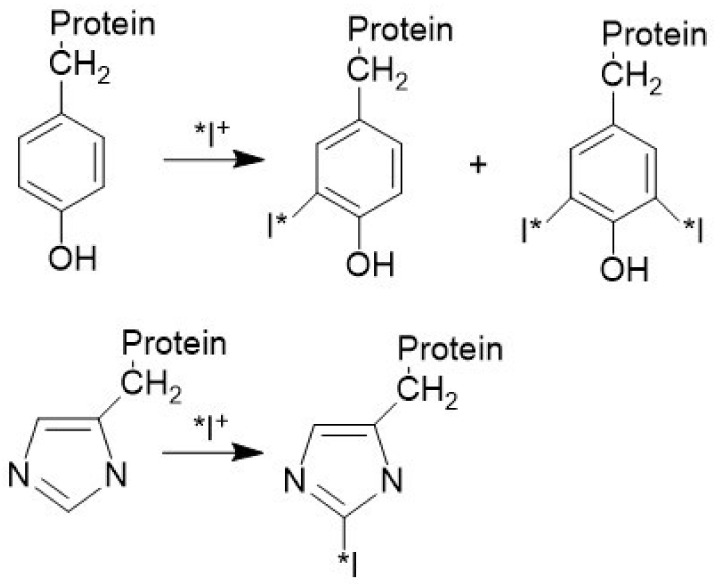
Radioiodination reactions of tyrosine and histidine residues in proteins.

The basic radioiodination reactions are shown in Figure 6. The positive radioactive iodine species (I^+^) generated in situ from the oxidation of radioiodide react with tyrosine and to some lesser extent to the histidine residues in the protein. Studies on the mechanism of the reaction of iodine with tyrosine and other phenols in stoichiometric amounts indicate that it is the phenolate anion which is radioiodinated. It is established that the primary site of the iodine addition is tyrosine amino acid residue in the large biomolecule; however, if the pH exceeds 8.5, the secondary site on the imidazole ring of histidine is favored. The oxidized I^+^ electrophilic species hydrolyze rapidly in aqueous solution forming the hydrated iodonium ion, H_2_OI^+^, and/or hypoiodous acid, HOI. With tyrosine, the substitution of a hydrogen ion with the reactive iodonium ion occurs *ortho*- to the phenolic hydroxyl group. Mono and di-iodination of tyrosine residue are observed. With histidine, substitution occurs at the 2-position of the five-member imidazole ring. Following the desired reaction period, residual reactive I^+^ species are reduced back to the I^−^ form and removed from the reaction solution by passage through either an anion exchange resin column or a gel filtration column. In this manner, high radiochemical purity can be achieved even if the labeling efficiency is low.

#### 4.1.1. Inorganic Oxidizing Agents Solution–Solution Phase Reactions

Numerous oxidizing reagents have been used for the direct radioiodination of proteins. Radioactive molecular Iodine was used as a labeling reagent in the early days of protein labeling. Since radioactive iodine is usually available as sodium iodide, Pressman and Keighly [36] used a mixture of ^131^I and I_2_ for the radioiodination of the protein. Later on, different oxidizing agents, e.g., sodium hypochlorite [107], nitrous acid [108,109] ammonium persulfate [110], hydrogen peroxide [111], ferric sulfate [112], and iodate [113] were used to generate radioactive molecular iodine before protein radiolabeling. Using molecular iodine as a radioiodination agent has some limitations, including: (1) the 50% maximum radiochemical yield and challenging purification. This is due to 50% conversion of iodide to iodine; (2) loss of radioactivity and increased exposure to the investigator due to volatility of molecular iodine; and (3) lower molar activity.

A technique using Iodine Monochloride (ICl), which eliminated the limitation of using molecular iodine, for protein radioiodination was developed [114,115,116]. The iodine-chlorine bond in ICl is polarized with a partial positive charge on the iodine, so the radiochemical yield is potentially 100%. The reagent was prepared by treating unlabeled ICl with radioactive sodium iodide. ICl, in the form of ICl_2_^−^ is prepared from the reaction of sodium iodide and NaIO_3_ in an acidic medium [117,118]. Studies on the iodination of phenol and substituted phenols with unlabeled ICl suggested a mechanism involving the electrophilic attack of iodide on the phenoxide ion followed by a slow loss of a proton. The electrophilic species has been suggested to be H_2_OI^+^ at low pH and ICl at higher pH or HOI [119,120].

More recently, a simple and rapid non-radioactive/radioactive iodide labeling method for peptides and proteins was developed [121,122]. In the method inorganic oxidizing agents, Hypochlorous acid/Hypochlorite and inorganic chloramines (NH_2_Cl, NHCl_2_, and NCl_3_) were used to generate iodine monochloride in situ for radioiodination of a tyrosine residue in peptides and proteins. The radiolabeling yields were high with >99% radiochemical purity.

#### 4.1.2. Organic Oxidizing Reagents for Solution–Solution Phase Reactions

**Figure 7:**
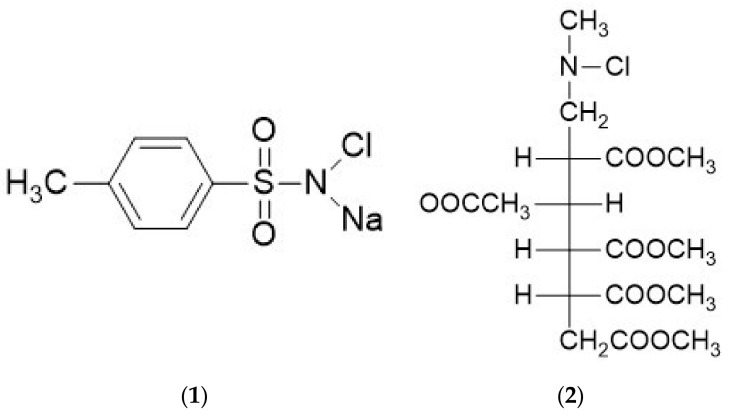
Structures of Chloramine-T (**1**) and Penta-*O*-acetyl-*N*-chloro-*N*-methylglucamine (NCMGE) (**2**).

The most widely used reagent for the radioiodination of peptides and proteins is chloramine-T, the sodium salt of N-monochloro-*p*-toluene-sulfonamide (Figure 7, Structure **1**), developed by Hunter and Greenwood [123,124]. In an aqueous solution, it forms HOCl, which is thought to be the actual oxidizing species [125]. This reacts with the radioactive iodide present to form an electrophilic iodine species, possibly H_2_OI^+^. On the other hand, the reaction of N-chloro derivatives with iodide was proposed via Cl^+^ atom transfer or formation of an association complex to form ICl [126,127]. Only a few micrograms of Chloramine-T and a short reaction time are required to achieve nearly quantitative iodination of proteins as it is a very effective oxidizing agent. Longer reaction times may cause significant damage to the protein, including thiol oxidation, chlorination of aromatic rings and/or primary amines, and peptide bond cleavage [105]. In a typical radioiodination experiment, the protein solution is mixed with radioactive iodide in a slightly alkaline buffer (pH 7.5) and a freshly prepared solution of chloramine-T. The reaction mixture is incubated at room temperature for a specified time optimized for the reaction. At the end of the incubation period, a slight molar excess of reducing agent, sodium metabisulfite (Na_2_S_2_O_5_), is immediately added to the mixture to reduce and inactivate the chloramine-T. It is important to note that the reducing agent, Na_2_S_2_O_5_, used for reaction quenching can also cause cleavage of the disulfide bridges within the protein molecules and alter the tertiary structure of the protein.

N-chloro derivatives of secondary amines with lower oxidation potential, instead of chloramine-T, were used for radio iodination to reduce the oxidative damage to proteins [128]. For example, N-chloro-morpholine was found to produce higher radioiodination yields and less degradation than chloramine-T when reacting with l-tyrosine or leucine enkephalin (Leu-Gly-Gly-Phe-Leu) [129,130]. In situ preparation of the fresh reagent was required due to its instability. A water-soluble low oxidation potential reagent, Penta-*O*-acetyl-*N*-chloro-*N*-methylglucamine (NCMGE) (Figure 7, Structure **2**) was found to be stable, producing higher radiochemical yield, and less decomposition to model amino acids and small peptides than chloramine-T [131,132].

#### 4.1.3. Organic Oxidizing Reagents for Solid–Solution Phase Reactions 

**Figure 8:**
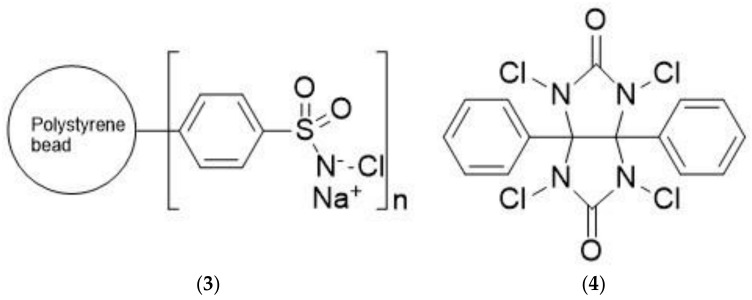
Structures of IODO beads (Chloramine-T attached to polystyrene bead, (**3**), and Iodogen (1,3,4,6-Tetrachloro-3α,6α-diphenyl-glycoluril, (**4**).

To minimize the oxidative damage to substrates caused by the chloramine-T/sodium metabisulfite method, a technique was developed by controlling the release of chloramine-T during the radio iodination reaction. This is accomplished by using the covalently attached chloramine-T to the surface of ~3 mm-diameter polystyrene beads with 0.55 ± 0.05 µmole/bead oxidizing capacity, known as IODO beads. (Figure 8, Structure **3**) These beads can be easily removed from the reaction mixture with a tweezer or by decanting the solution to stop the radio iodination reaction [133]. These beads are commercially available from Thermo Fisher Scientific. The technique has the following advantages: (1) the rates of radio iodination and oxidative damage of protein are slow as the reaction occurs on the solid surface rather than in solution; (2) the oxidative damage of the protein is low, but not eliminated; (3) reductive damage caused by metabisulfite is eliminated, as there is no need to stop the reaction chemically; and (4) the IODO beads are commercially available.

Radioiodination, using IODO beads, is accomplished by adding the buffered protein solution to a test tube containing IODO beads followed by the desired amount of sodium radioiodide solution. The rate of the reaction in the presence of IODO beads is slower than that with soluble chloramine-T and labeling efficiencies are somewhat lower for the same reasons. At the end of the incubation period, the IODO beads are removed and the reaction mixture is transferred to a gel column for purification.

A new reagent, 1,3,4,6-tetrachloro-3α, 6α-diphenyl glycoluril (Iodogen, Figure 8, Structure **4**), was introduced to iodinate proteins and to minimize their damage during radioiodination [134,135]. This reagent has several advantages: (1) Iodogen is virtually insoluble in aqueous media; the protein solution does not form a homogenous solution with the oxidizing agent; and (2) the radio iodination reaction could be stopped by simply removing the sample solution from the reaction tube, thus avoiding any use of reducing agent. Several proteins were radioiodinated by using the Iododen method [136] and the properties of the iodinated proteins were unaltered as confirmed by gel filtration, isoelectric focusing, and immunological reactivity. The stability of the labeled proteins during storage was good.

The Iodogen reagent is supplied by Thermo Fisher Scientific in the powder form. Iodogen-coated tubes can be prepared in advance by transferring aliquots of 20 µL (0.1 mg/mL concentration) Iodogen solution in methylene chloride into the suitable glass or polypropylene tubes. The tubes are allowed to dry under nitrogen at room temperature. Alternatively, the pre-coated Iodogen tubes, ready for single-use, can be purchased from Thermo Fisher. Each 12 × 75 mm tube is coated with ~50 µg of Iodogen at the bottom. The radioiodination procedure involves the same steps as for the IODO beads method. Variable rates of the radioiodination of proteins were observed depending on the solid surface on which Iodogen was coated [137]. For example, polypropylene test tubes resulted in the lowest rate of oxidation, followed by borosilicate glass, with polar soda-lime glass giving the highest rate of oxidation.

#### 4.1.4. Enzyme Catalysts for Solution–Solution Phase Reactions

Some enzymes, e.g., peroxidases (lactoperoxidase, horseradish peroxidase, myeloperoxidase, and chloroperoxidase) are known for catalyzing the mild oxidation of iodide, in the presence of nanomolar concentrations of hydrogen peroxide (H_2_O_2_), for radioiodination of tyrosine, and to some extent histidine also, in proteins. The most extensively used enzyme is Lactoperoxidae for the radioiodination of proteins in the past. Hydrogen peroxide itself is capable of oxidizing radioiodide followed by radioiodination of proteins. Lactoperoxidase can also radioiodinate histidines in the proteins; however, the rate of iodination of histidines is much slower than the rate of iodination of tyrosines [138,139]. Lactoperoxidase is used as a catalyst, for peroxide oxidation of iodide, which permits extremely low H_2_O_2_ concentrations to be used [140]. In a typical radioiodination experiment, 2 to 10 µg protein are mixed with 1 to 10 mCi of radioiodide and 20 to 100 ng of lactoperoxidase. The reaction is initiated by the addition of 50 to 100 ng of H_2_O_2_ followed by the addition of 30 to 50 ng of H_2_O_2_ at 10 to 15 min intervals. After 30 to 60 min of incubation at room temperature, the reaction is quenched by the addition of cysteine or by dilution. Free iodide is removed by gel filtration or by other procedures. The rate of radioiodination is dependent on pH. Generally, a pH of 5.6 was found to be the optimal pH in most radiolabeling experiments. The immunological and biological properties of the original biomolecule are maintained as it is not exposed to strong oxidizing and reducing agents [141].

During radioiodination, the lactoperoxidase, containing 15 tyrosine and 14 histidine residues, is self-iodinated, leading to the loss of iodide and challenging separation. This problem was solved by attaching lactoperoxidase to Sephadex beads (Enzymobeads). In the labeling procedure, the Enzymobeads first were hydrated in distilled water for 2 to 4 h before use. 50 to 100 µg protein in 0.2 M phosphate buffer (50 µL, pH 7.2) was mixed with the Enzymobeads (25 µL suspension), 1 to 5 mCi of radioiodide, and 25 µL of 1% β-d-glucose. The radioiodination was allowed to proceed at room temperature for 15 to 25 min. The enzymobeads were removed by centrifugation or membrane filtration. The free iodide was removed by gel filtration or dialysis.

Bio-Rad Laboratories developed a novel commercial solid-state system, insoluble resin beads were covalently coated with a mixture of two enzymes: lactoperoxidase and glucose oxidase. When buffered solutions of protein and radioiodide were added to a suspension of the enzyme-coated beads in the presence of a small quantity of glucose, a chain of events was initiated: (1) the glucose oxidase enzyme used the glucose to generate a small amount of hydrogen peroxide at the surface of the beads; (2) the lactoperoxidase attached to the beads catalyzed the oxidation of iodide by the generated H_2_O_2_ in the solution; and (3) oxidized iodide radioiodinated the tyrosine residues in the protein. The reaction mixture was separated from the beads by decanting or centrifuging followed by loading onto a gel filtration column. Upon column elution, the desired labeled protein eluted first, the unreacted radioiodine was retained within the gel. BioRad, unfortunately, stopped supplying these beads in the early 1990s.

### 4.2. Indirect Labeling Methods 

Sometimes it is not possible to radioiodinate proteins by direct electrophilic addition to tyrosine and histidine residues. This may be due to the fact that (1) a limited number of tyrosine and histidine residues may be present in the protein; (2) these may be buried within the tertiary structure of the protein and may not be readily available for radioiodination; and (3) these may be located at or near the active binding site of the molecule which cannot be disturbed. Consequently, several other labeling strategies have been developed to radioiodinate protein molecules at sites other than tyrosine and histidine.

A most common alternative approach is using a prosthetic group for radiolabeling of proteins. A prosthetic group for radioiodination contains an aromatic moiety, like tyrosine, which can be iodinated and covalently attached to the lysine moiety in the protein [142,143,144]. Two methods can be employed for the radioiodination of a protein. In the first method, the prosthetic group is radioiodinated, by using the methods given above, followed by coupling with the protein, thereby avoiding exposure of sensitive functionalities in the target molecule to oxidation. The coupling reaction must be efficient to avoid loss of radioiodide. In the second method, the prosthetic group is coupled with the protein followed by radioiodination using one of the methods given above. Overall radiolabeling efficiencies are lower with this approach for the simple reason that two separate labeling reactions are used. The main issue with this technique is to ensure the preservation of the immunological and biological properties of the protein. An early example of the use of prosthetic groups for radioiodination was the treatment of insulin with 4-[^131^I]iodobenzenediazonium chloride [145].

**Figure 9:**
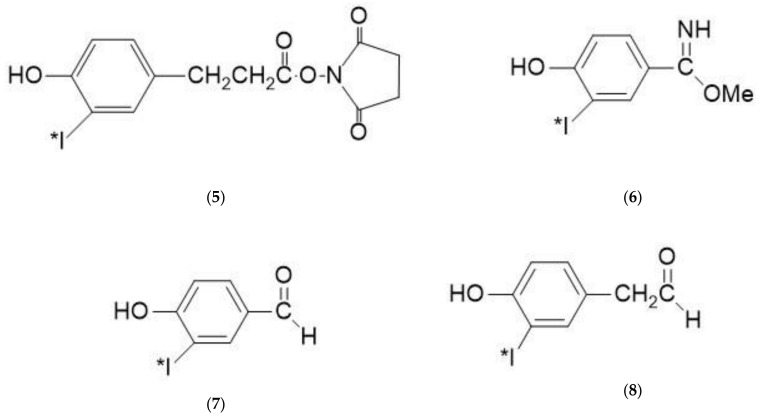
Structures of *N*-hydroxysuccinimide ester of 3-(4-Hydroxyphenyl) propionic acid (Bolton Hunter reagent, **5**), *p*-Hydroxybenzimidate (Wood’s reagent, **6**), *p*-Hydroxy benzaldehyde (**7**), and *p*-Hydroxybenzaacetaldehyde (**8**).

The fact that some proteins are sensitive to oxidation and lack tyrosine for radioiodination prompted Bolton and Hunter to develop a reagent, *N*-hydroxysuccinimide Ester of 3-(4-Hydroxyphenyl) Propionic Acid [146] (Figure 9, structure **5**) which could be conjugated to a protein under milder conditions than those found in direct radioiodinations. Other reagents such as *p*-Hydroxybenzimidate (Wood’s reagent) [147] (Figure 9, Structure **6**), *p*-Hydroxybenzaldehyde [148] (Figure 9, Structure **7**), and *p*-Hydroxybenzacetaldehyde [149] (Figure 9, Structure **8**) have also been studied as radioiodination reagents.

## 5. Overview of ImmunoPET Imaging Pharmaceuticals for Cancer-Preclinical

PET imaging pharmaceuticals are routinely used for early detection of cancer and monitoring the progress of treatment following surgery, chemotherapy, and radiotherapy [150]. Numerous ^124^I-labeled small molecules have been produced by nucleophilic and electrophilic substitution reactions and tested for various targets. Some of the ^124^I-labeled PET imaging pharmaceuticals based on the small molecule (with the target given in the parenthesis) are ^124^I-MIBG (adrenergic activity), ^124^I-IAZA and ^124^I-IAZG (hypoxia agent), ^124^I-dRFIB, ^124^I-IUdR and ^124^I-CDK4/6 inhibitors (cell proliferation), ^124^I-hypericin (protein-kinase C), ^124^I-FIAU (herpes virus thymidine kinase), *m*-^124^I-IPPM (opioid receptors), ^124^I-IPQA ((EGFR kinase activity), ^124^I-labeled-6-anilino-quinazoline (EGFR inhibitors), ^124^I-purpurinimide (tumor imaging) [151].

As mentioned above, ImmunoPET is a paradigm-shifting molecular imaging modality that involves a combination of targeting specificity of mAbs and the high sensitivity of the PET imaging technique [14,152,153]. ImmunoPET imaging provides excellent specificity, sensitivity, and resolution in detecting primary tumors and is the method of choice for imaging specific tumor markers, immune cells, immune checkpoints, and inflammatory processes. Various ^124^I-labeled antibodies, nanobodies, antibody fragments, and proteins have been used for molecular imaging of differentiated thyroid cancer, breast cancer, colorectal cancer, clear-cell renal cell carcinoma, ovarian cancer, and neuroblastoma, etc. Clinical feasibility of ^124^I-labeled mAb (HMFGI) as an immunoPET imaging pharmaceutical, for quantitative measurement of distribution and blood flow in breast cancer patients by using ^124^I and PET, was first demonstrated in 1991 [154]. Herein, we present an overview of the development strategies for target-specific ^124^I-labeled ImmunoPET imaging pharmaceuticals and their preclinical and clinical applications over the past three decades.

### 5.1. Receptor Tyrosine Kinase

Receptor tyrosine kinases (RTKs) are high-affinity cell surface receptors which play an important role in a variety of cellular processes, including growth, motility, differentiation, and metabolism. RTKs are key regulators of normal cellular processes with a critical role in the development and progression of many types of cancers [155]. Approximately 20 different RTK classes have been identified, including the Epidermal Growth Factor Receptor (EGFR) family which includes HER1 (ErbB1), HER2 (Neu, ErB2), HER3(ErbB3), and HER4 (Erb4) and Vascular Endothelial Growth Factor (VEGFR). Two RTKs (EGFR and VEGFR) have been targeted the most for the development of immunoPET imaging pharmaceuticals.

#### 5.1.1. Epidermal Growth Factor Receptor (EGFR)

The Epidermal Growth Factor Receptor, a transmembrane protein, is highly expressed in a variety of human cancers, including non-small-cell lung cancer (NSCLS). The overexpression of EGFR has been observed in both premalignant lesions and malignant tumors of the lung, in 40–80% patients with NSCLS, in 18–25% of all breast cancer carcinoma (specifically HER2 expression), and subsets of ovarian, lung, prostate and gastric cancers [156,157]. Breast cancers overexpressing HER2 have been associated with aggressive tumor growth, high relapse, poor prognosis, and being more resistant to endocrine therapy and chemotherapy. Consequently, substantial research has been conducted in the development of immunoPET imaging approaches and pharmaceuticals for the evaluation of the heterogeneous status of RTKs in cancers [158].

Trastuzumab (Herceptin), Cetuximab (Erbitux), Panitumumab (Vectibix), and Nimotuzumab (BioMAb) have been approved, recently, for the treatment of EGFR positive cancers by targeting the extracellular domain of EGFR. PET and SPECT techniques using radiolabeled antibodies, including trastuzumab, pertuzumab, and trastuzumab fragment, were able to detect HER2 expression; however, their large size resulted in slow tumor uptake and clearance from circulation [159,160].

Several ^124^I-labeled mAbs have been investigated as potential immunoPET imaging pharmaceuticals for targeting a wide variety of tumors overexpressing the human EGFR. For example, ^124^I-labeled ICR 12, a rat mAb recognizing the external domain of the human c-Erb B2 protooncogene, was evaluated as a potential PET imaging pharmaceutical for breast cancer patients. Biodistribution and imaging studies were performed in athymic mice bearing human breast carcinoma xenografts. Good tumor uptake (up to 12% ID/g at 120 h post-injection), with localization indices (3.4–6.2) was observed. Tumor xenografts of 6 mm diameter were successfully imaged with high resolution at 24, 48, and 120 h post-injection [161]. Two ^124^I-labeled mAbs, MX35 and MH99, were also evaluated in nude rats bearing subcutaneous human SK-OV-7 and SK-OV-3 ovarian cancer xenografts. A melanoma cell line (SK-MEL-30) was used as a negative control tumor. Subcutaneous ovarian cancer nodules as small as 7 mm were identified with PET imaging. Tumor uptake was seen as high as six times to the normal tissue [162].

^124^I-labeled C6.5 diabody, a small-engineered antibody fragment that is specific for the HER2 receptor tyrosine kinase, was investigated by using SCID mice bearing HER2-positive human ovarian carcinoma (SK-OV-3) xenografts. The diabody accumulated in SK-OV-3 tumors and blood at 48 h post-injection [163]. ^124^I-labeled mAb, Trastuzumab, and a small 7-kDa scaffold protein, the affibody molecule, were evaluated and compared for the development of anti-HER2 targeting immunoPET imaging pharmaceuticals [164]. Both moieties were found to bind with HER2-expressing cells in vitro and xenografts in vivo. Total uptake of trastuzumab in tumors was higher than that of ^124^I-labeled affibody. However, tumor-to-organ ratios were appreciably higher for ^124^I-labeled affibody due to its more rapid clearance from blood and normal organs. A small-animal study was used to confirm ex vivo results. The study concluded that the use of the small scaffold targeting affibody provides better contrast in HER2 imaging than does the mAb.

A fragment of Trastuzumab (Fab) was modified via PASylation (PAS = Pro-Ala-Ser chain) for blood circulation optimization, radiolabeling with ^89^Zr and ^124^I, and their comparative performance assessment in CD1-*Foxn1^nu^* mice bearing HER2-positive xenografts. The ^89^Zr- and ^124^I-labeled Fab-PAS_200_ showed specific tumor uptakes of 11% ID/g and 2.3% ID/g 24 h post-injection with high contrast, respectively, with high tumor-to-blood (3.6 and 4.4) and tumor-to-muscle (20 and 43) ratios [165].

Several mouse mAbs have been screened in the past and it was found that mAb 806 specifically targets the overexpressed or activated forms of EGFR [166,167]. ch806, a chimeric form of mAb 806 which has been validated as an effective therapeutic antibody, showed specific accumulation of the antibody at multiple tumor sites and potential for molecular imaging. Biodistribution studies, in BALB/c nude mice bearing de2–7 EGFR-expressing xenografts, revealed that ^125^I-labeled ch806 did not show significant tumor retention. However, specific and prolonged tumor localization of ^111^In-labeled ch806 was demonstrated with the uptake of 31% ID/g and a tumor to blood ratio of 5:1 observed at 7 days post- injection [168].

The chimeric antibody, ch806, was conjugated with the residualizing ligand IMP-R4 for ^124^I labeling, in vivo biodistribution, and small-animal PET imaging studies in BALB/c nude mice bearing U87MG.de2–7 glioma xenografts. The biodistribution data analysis showed 30.95 ± 6.01% (% ID/g) tumor uptake of ^124^I-IMP-R4-ch806 injected dose at 48 h post-injection, with prolonged tumor retention (6.07 ± 0.80%I D/g at 216 h post-injection). The tumor-to-blood ratio increased from 0.44 at 4 h post-injection to a maximum of 4.70 at 168 h post-injection. PET images of ^124^I-IMP-R4-ch806 were able to detect the U87MG.de2-7 tumors at 24 h post- injection and for at least 168 h post-injection [169]. Similarly, the mean uptake of ^124^I-PEG4-tptddYddtpt-ch806 by U87MG.de2-7 glioma xenografts reached a maximum of 36.03% ±5.08% ID/g at 72 h post injection. These studies suggest that the chimeric antibody, ch806, has potential for further studies [170].

#### 5.1.2. Vascular Endothelial Growth Factor (VEGF)

Vascular endothelial growth factor (VEGF) is a signal protein produced by cells that stimulates the formation of blood vessels. They are important signaling proteins involved in both vasculogenesis (the de novo formation of the embryonic circulatory system) and angiogenesis (the growth of blood vessels from pre-existing vasculature). The VEGF and its receptor (VEGFR) have been shown to play major roles not only in physiological but also in most pathological angiogenesis, such as cancer [171]. Several therapeutic agents targeting VEGF (e.g., bevacizumab and ramucirumab) and VEGFR (e.g., sorafenib and sunitinib) have been approved for clinical use around the world [172]. Clinical immunoPET studies using ^89^Zr-labeled bevacizumab were performed in a variety of tumors, including breast cancer [173], neuroendocrine tumors [174], renal cell carcinoma (RCC) [175], NSCLC [176], and glioma [177,178].

An IgG1 monoclonal antibody, VG76e, that binds to human VEGF, was labeled with ^124^I (i.e., [^124^I]-SHPPVG76e) and was investigated in the HT1080 human fibrosarcoma xenografts in immune-deficient mice for VEGF-specific localization. A single intravenous injection of [^124^I]-SHPPVG76e into tumor-bearing mice showed a time-dependent and specific localization of the tracer to the tumor tissue. High tumor-to-background contrast and distribution of [^124^I]-SHPPVG76e in the major organs were seen in the whole-body animal PET imaging studies. These studies support further development of [^124^I]-SHPP-VG76e as an immunoPET imaging pharmaceutical for measuring tumor levels of VEGF in humans [179].

### 5.2. Clusters of Differentiation

The clusters of differentiation (CD) antigens are cell-surface receptors involved in cellular functions like activation, adhesion, and inhibition. These receptors express elevated levels of the CD on cells which can serve as key markers in several cancers and infectious diseases. CD markers are mostly useful for classifying white blood cells (WBC) and especially important for the diagnosis of lymphomas and leukemias. The CD nomenclature was proposed and established a long time ago. Since then, its use has expanded to many other cell types, and more than 320 CD unique clusters and sub clusters have been identified.

Several CD antigens have been investigated as diagnostics or therapeutics targets in the past. For example, CD20 and CD30 are common biomarkers for lymphoma imaging [180,181,182] and the Food and Drug Administration approved a CD20-specific chimeric mAb, Rituximab, for the treatment of non-Hodgkin’s lymphoma (NHL) and rheumatoid arthritis (RA). Feasibility studies, related to ^64^Cu-DOTA-rituximab and ^89^Zr-labeled rituximab as immunoPET imaging pharmaceutical for CD20 expression in NHL-bearing humanized mouse models and translating later into the clinic, were reported followed by the translation of ^89^Zr-labeled Rituximab into the clinic [183,184,185].

#### 5.2.1. Cluster of Differentiation 20 (CD 20)

Tumor targeting of anti-CD20 diabodies (scFv dimers) for detection of low-grade B-cell lymphoma was investigated. The scFv-8 and Cys-Db were labeled with ^124^I and ^131^I for PET imaging and biodistribution, respectively, at 2, 4, 10, and 20 h. Mice bearing 38C13-huCD20 (positive) and wild-type 38C13 (negative) tumors were used. Both ^124^I-labeled scFv-8 and Cys-Db exhibited similar tumor targeting at 8 h post-injection, with significantly higher uptakes than in control tumors. At 20 h, less than 1% ID/g of ^131^I-labeled Cys-Db was present in tumors and tissues [186]. Two recombinant anti-CD20 rituximab fragments, a minibody, Mb (scFv-C_H_3 dimer; 80 kDa) and a modified scFv-Fc fragment (105 kDa), designed to clear rapidly, were produced and labeled with ^64^Cu and ^124^I. Rapid and specific localization to CD20-positive tumors was observed with both radioiodinated fragments producing high-contrast images in vivo. The ^124^I-labeled mini body showed higher uptake in CD-20 positive tumors than scFv-Fc [187].

In yet another similar report, cys-diabody (cDb) and cys-mini body (cMb) based on rituximab and obinutuzumab (GA101) were labeled with ^124^I and used to target the CD20 antigen in transgenic mice and a CD20-expressing murine lymphoma model. Obinutuzumab-based imaging pharmaceuticals (^124^I-GAcDb and ^124^I-GAcMb) produced high-contrast immunoPET images of B-cell lymphoma and outperformed the respective rituximab-based tracers [188].

#### 5.2.2. Cluster of Differentiation 274 (CD274)

The Cluster of differentiation 274, CD274, or Programmed Death-Ligand 1 (PD-L1), is a protein (40 kD type 1 transmembrane protein) that in humans is encoded by the CD274 gene. Upregulation of PD-L1 may allow cancers to evade the host immune system. An analysis of 196 tumor specimens from patients with renal cell carcinoma found that high tumor expression of PD-L1 was associated with increased tumor aggressiveness. Many PD-L1 inhibitors, durvalumab, pembrolizumab, atezolizumab, and avelumab, are in development as immuno-oncology therapies and are showing good results in clinical trials.

A novel heavy-chain antibody (HCAb) was constructed and labeled with ^124^I to target the programmed cell death ligand-1 (hPD-L1) which is known to activate T cells associated with malignancies. Biodistribution studies in osteosarcoma OS-732 tumor-bearing mouse model showed a tumor uptake of 4.43 ± 0.33% ID/g at 24 h. Tumor lesions were detected on micro PET/CT 24 h post-injection [189]. In continuation for development of ^124^I-labeled imaging pharmaceuticals, JS001 (Toripalimab, a humanized IgG mAb) was investigated for targeting human PD-L1 (hPD-L1) in a tumor mouse model [190].

### 5.3. Carbohydrate Antigen

Carbohydrate antigen 19-9 (CA19-9), also known as sialyl-Lewis A, is a tetrasaccharide that is usually attached to O-glycans on the surface of cells. It is known to play a vital role in cell-to-cell recognition processes [191] and as an established biomarker for several cancers, including, lung, breast, and PDAC (Pancreatic Ductal Adenocarcinoma). CA19-9 is the most highly expressed tumor antigen, present on cellular membrane proteins in more than 90% of pancreas cancer patients [192]. 5B1, a fully human IgG monoclonal antibody, is a known anti-CA 19-9 antibody that has been used as a theranostic agent [193,194,195]. For example, in a first-in-human clinical trial, ^89^Zr-labeled 5B1 was used for immunoPET imaging of detected known PDACs, metastases [196].

On the contrary, Girgis and coworkers created several antibodies and diabodies for targeting CA 19-9 antigens expressed by pancreas cancer. An anti-CA19-9 monoclonal antibody and a cys-diabody, created by engineering C-terminal cysteine residue into the DNA single-chain Fv construct of CA19-9, were labeled with ^124^I and injected into mice harboring CA19-9 antigen-positive and CA19-9 negative xenografts. MicroPET/CT imaging was performed at 72, 96, and 120 h post-injection. The average tumor to blood (% ID/g) ratio was 5.0 and 3.0 and the average positive tumor to negative tumor (% ID/g) ratio was 20.0 and 7.4 for mAb and cys-diabody, respectively [197,198]. Another diabody (~55 kDa) construct, which was created by isolation of variable region genes of the intact anti-CA 19-9 antibody, was created, ^124^I-labeled, and tested in mice harboring an antigen-positive (BxPC3 or Capan-2) and a negative xenograft (MiaPaca-2). Pancreas xenograft imaging of BxPC3/MiaPaca-2 and Capan-2/MiaPaca-2 models with the anti-CA19-9 diabody demonstrated an average tumor: blood ratio of 5.0 and 2.0, respectively, and an average positive: negative tumor ratio of 11 and 6, respectively [199].

An Fc-mutated, anti-CA19-9 antibody fragment, scFv-Fc H310A, 105 kD dimer, was created for microPET imaging of pancreatic cancer xenografts. The ^124^I-scFv-Fc H310A localized to the antigen-positive tumor xenografts and confirmed by microPET imaging. Higher % ID/g in the antigen-positive tumor compared to the blood, antigen-negative tumor, and liver was observed [200]. ^124^I-JAA-F11 was investigated to target Thomsen–Friedenreich antigen (TF-Ag), a mucin-type disaccharide galactose-b1-3*N*-acetylgalactosamine conjugated to proteins by an alpha-*O*-serine or *O*-threonine linkage, which is found on human carcinomas of many types including those of the breast, colon, bladder, and prostate [201].

### 5.4. Carcinoembryonic Antigen (CEA)

Carcinoembryonic antigen (CEA), a protein, is normally present at very low levels in the adult blood but may be elevated with certain types of cancers. CEA serves as a vital tumor antigen and a serum tumor marker [202]. The normal CEA range in adult’s blood is <2.5 ng/mL (non-smoker) and <5.0 ng/mL (smoker) and is elevated in cancer patients. The most common cancers that show elevated CEA levels are colon, rectum, and ovarian. Arcitumomab (CEAScan) is a ^99m^Tc-labeled hapten peptide pre targeted imaging probe approved by the FDA and EMA (European Medicine Agency) for detecting colonic cancer metastases [203]. 

A series of antibody fragments were engineered from a murine mAb, T84.66, and have been radiolabeled with ^64^Cu and ^124^I [204,205]. The ^124^I-labeled anti-CEA T84.66 mini body (single-chain Fv fragment [scFv]-C(H)_3_ dimer, 80 kDa) and diabody (noncovalent dimer of scFv, 55 kDa) were evaluated in the athymic mouse/LS174T xenograft model PET images, 18 h post injection, using mini body and diabody, showed specific binding to the CEA-positive xenografts and relatively low activity in normal tissues. Target to background (T/B) ratios were 3.05, 3.95 and 11.03, and 10.93 at 4 and 8 h post injection for mini and diabody, respectively [205]. To improve the T/B ratio of the engineered antibody fragments, mutation of the residues in the Fc fragment was performed. A series of anti-CEA scFv-Fc fragments were evaluated for tumor localization and pharmacokinetics [206,207] in LS174T xenografted athymic mice by small-animal PET. The PET imaging with a ^124^I-labeled scFv-Fc with double mutation (H310A/H435Q) quickly localized to the tumor site, rapidly cleared from animal circulation, and produced clear images [207].

A pretargeting technique for targeting CEA expressing tumors was developed [208,209]. The pretargeting technique uses a bispecific monoclonal antibody bs-mAb (a multivalent, recombinant anti-CEA, carcinoembryonic antigen/anti-HSG histamine-succinyl-glycine fusion protein) with the affinity for a tumor and a small hapten peptide. Typically, mice are implanted with CEA-expressing LS174T human colonic tumors, a bispecific monoclonal anti-CEA/anti-HSG/anti-hapten antibody is given to the mice, followed by an administration of a radiolabeled hapten peptide. A new peptide, IMP-325, In-DOTA-d-Tyr-d-Lys(HSG)-d-Glu-d-Lys(HSG)-NH_2_, was labeled with ^124^I and tested in nude mice bearing LS174T human colonic tumors that were given anti-CEA/anti-HSG bs-mAb. The ^124^I-IMP-325 alone cleared quickly from the blood with no evidence of tumor targeting, but when pretargeted with the bs-mAb, tumor uptake increased 70-fold, with efficient and rapid clearance from normal tissues, allowing clear visualization of the tumor within 122 h [210].

### 5.5. Carbonic Anhydrase IX

Carbonic Anhydrase IX is a transmembrane protein that is overexpressed in clear cell renal cell carcinoma (ccRCC) and carcinomas of the uterine cervix, kidney, esophagus, lung, breast, colon, brain, and hypoxic solid tumors. Its overexpression in cancerous tissues compared to normal ones is due to hypoxic conditions in the tumor microenvironment. Consequently, it is a cellular biomarker of hypoxia [211].

A chimeric mAb, cG250, subclass IgG1, was reported in 1986 to recognize an antigen which preferentially expresses on cell membranes of renal cell carcinoma (RCC). Since that time, G250 has been shown to localize in primary (98%) and metastatic (88%) ccRCC lesions found on human histologic slides under light microscopy [212]. Oxygen tension measurements were used to investigate hypoxia and carbonic anhydrase IX expression, tumor uptake, and biodistribution, in a renal cell carcinoma SK-RC-52 xenograft model using ^124^I-labeled cG250 and PET/CT. Oxygen tension was found to be significantly higher in normal tissues than in the xenograft tumor. Biodistribution studies of ^124^I-cG250 demonstrated isotope uptake in the xenografts peaking at 23.45 ± 5.07% ID/g at 48 h post-injection [213]. ^89^Zr-labeled, an alternative to ^124^I, cG250 was evaluated in ccRCC xenograft models in mice. Greater uptake, retention, and superior PET images for ^89^Zr-labeled cG250, due to trapping inside the tumor cell, compared to ^124^I–labeled cG250, due to internalization and release of ^124^I, were observed [214,215].

### 5.6. Glycoproteins

#### 5.6.1. Glycoprotein A33

The glycoprotein A33, GPA 33, is a transmembrane glycoprotein with homology to the immunoglobulin superfamily. This antigen is expressed in >95% of colorectal cancer and a subset of gastric and pancreatic cancers [216]. It contains three distinct structural domains: a 213 amino acid extracellular region containing two immunoglobulin-like domains, a 23 amino acid hydrophobic transmembrane domain, and a highly polar 62 amino acid intracellular tail containing four consecutive cysteine residues. ^125^I- and ^131^I-labeled murine mAb have been investigated as SPECT and Radioimmunotherapy agents, respectively, in phase I/II clinical trials [217,218].

A recombinant humanized anti-colorectal cancer A33 antibody, huA33, was labeled with ^124^I and used for biodistribution properties and PET imaging characteristics in SW1222 colorectal xenograft bearing BALB/c nude mice. Excellent tumor uptake, with a maximum of 50.0 ± 7.0% ID/g at 4 days post injection, was observed [219].

#### 5.6.2. Glycoprotein CD44v6

When the CD44 gene is expressed, its pre-messenger RNA (mRNA) can be alternatively spliced into mature mRNAs that encode several CD44 isoforms. The mRNA assembles with ten standard exons, and the sixth variant exon encodes CD44v6, which engages in a variety of biological processes, including cell growth, apoptosis, migration, and angiogenesis Overexpression of the mature mRNA encoding CD44v6 can induce cancer progression. For example, CD44v6 assists in colorectal cancer stem cells in colonization, invasion, and metastasis [220]. CD44v6 is also expressed in thyroid carcinoma on the outer cell surface of squamous-cell carcinomas of head-and-cancer [221]. U36, an anti-CD44v6 chimeric (mouse/human) monoclonal antibody (cmAb), was found to target CD44v6 antigen. Biodistribution and scintigraphy studies in nude mice bearing tumors from the HNX-OE human head and neck tumor cell line were conducted. Co-injection of ^124^I-cMAb U36 and ^131^I-cMAb U36 provided similar tissue uptake values. Selective tumor uptake was confirmed with PET imaging at 24, 48, and 72 h post injection, which detected 15 out of 15 tumors [222]. A comparative biodistribution study of ^89^Zr- and ^124^I-labeled head and neck squamous cell carcinoma (HNSCC)-selective cMAb U36 versus ^88^Y-, ^131^I-, and ^186^Re-labeled cMAb U36 conjugates was conducted in HNSCC xenograft bearing mice at 24, 48, and 72 h post injection. Tumor uptake was higher for the ^89^Zr- and ^88^Y-labeled cMAb U36 than the ^124^I-, ^131^I-, and ^186^Re-labeled cMAb U36 [223]. Biodistribution and PET imaging studies of ^124^I-cMAb U36 nude mice bearing KAT-4 tumors in the left flank and the right front leg were performed. ^124^I-cmAb U36 uptake (%ID/g) in the flank tumors was 8.2 ± 3.6, 13.7 ± 0.7, 21.8 ± 2.8%, and 12.8 ± 5.2 at 4, 24, 48, and 72 h post injection, respectively. The tumors were visible in PET images at all-time points with the highest uptakes at 48 h post injection [224].

### 5.7. Prostate-Specific Membrane Antigen (PSMA)

Prostate cancer (PCa) is the most common cancer in men [225]; therefore, early detection of primary disease and its metastases is critical for clinical staging, prognosis, and therapy management. The prostate-specific membrane antigen (PSMA) is a transmembrane glycoprotein that is significantly over-expressed in most early-stage prostate cancer cells compared to benign prostatic tissues. Consequently, it has gained significant interest as a target for imaging and therapy in the past five years [226,227].

Capromab pendetide (ProstaScint^®^) is the murine mAb, 7E11-C5.3, conjugated to the DTPA chelator. The 7E11-C5.3 antibody is of the IgG1, kappa subclass (IgG1κ). This antibody is directed against Prostate-Specific Membrane Antigen (PSMA). ^111^In-labeled Capromab pendetide is approved by the FDA for prostate cancer imaging in newly-diagnosed patients by biopsy. ^124^I-labeled Carpromab was proposed as a PET imaging pharmaceuticals to decrease the retention of radioactivity in healthy organs, due to the non-residualizing properties of the radiolabel. Carpromab was radioiodinated and its targeting properties were compared with the ^111^In-labeled counterparts in LNCaP xenografts. PSMA-negative xenografts (PC3) were used as the negative control. Biodistribution of ^125^I/^111^In-capromab showed more rapid clearance of iodine radioactivity from liver, spleen, kidneys, bones, colon tissue, as well as tumors. Maximum tumor uptake (13 ± 8% ID/g for iodine and 29 ± 9% ID/g for indium) and tumor-to-non-tumor ratios for both agents were measured at 5 days post-injection. High tumor accumulation and low uptake of radioactivity in normal organs were confirmed using micro PET/CT at 5 days post-injection of ^124^I-capromab. Although tumor uptake was relatively lower for the ^124^I-Capromab than ^111^In-Capromab in LNCaP xenografts (13 ± 8% vs. 29 ± 9% ID/g), it showed lower uptake in normal organs compared to its ^111^In counterpart [228]. More recently, Frigerio et al. demonstrated the targeting specificity and sensitivity of ^124^I-labeled anti-PSMA single-chain variable fragment (scFv) in a preclinical in vivo model. The uptake of ^124^I-scFv was found to be very high and specific for PSMA-positive cells [229]. J591, a humanized mAb that binds to an extracellular domain of PSMA, has been investigated for both imaging and therapy [230,231,232,233,234]. It was demonstrated recently that ^124^I- and ^89^Zr-labeled J591 had comparable surface binding and internalization rates in preclinical prostate models [235]. These studies imply that PCa theranostics using ^177^Lu- and ^124^I- or ^89^Zr- labeled J591 is feasible, safe, and may have superior targeting toward bone lesions relative to conventional imaging modalities.

### 5.8. Prostate Stem Cell Antigen (PSCA)

Prostate stem cell antigen (PSCA) is a protein that in humans is encoded by the PSCA gene. This gene encodes a glycosylphosphatidylinositol-anchored cell membrane glycoprotein. PSCA is expressed in 83%–100% of prostate cancers [236,237,238,239,240]. It is also highly expressed in most prostate cancer bone metastases (87–100%) and the local bladder, pancreatic carcinoma, bladder, placenta, colon, kidney, and stomach cancers [241,242,243,244].

The anti-PSCA murine mAb 1G8 showed anti-tumor activity [245]. An ^124^I-labeled 2B3 anti-PSCA minibody, a hu1G8 minibody fragment dimer of scFvs-C_H_3 with an 18 amino acids linker and ~80 kDa molecular weight, was evaluated in mice bearing LAPC-9 (PSCA-positive) and PC-3 (PSCA-negative) xenografts. Micro PET imaging of the PSCA positive tumors showed ^124^I-2B3 minibody to target and image PSCA-expressing xenografts with high contrast at earlier time points than the ^124^I-labeled intact hu1G8 anti-PSCA mAb. This was due to faster clearance of the minibody than the anti-PSCA mAb [246,247]. The parental 2B3 diabody (p2B3-Db) (molecular weight, 55 kDa) was back mutated with a linker of 8 amino acids to produce a high-affinity diabody (bm2B3-Db8). ^124^I-p2B3-Db8 and bm2B3-Db8 were evaluated in bio-distribution and for tumor imaging studies in nude mice bearing xenografts of the LAPC-9 and PC-3 (PSCA-negative) tumor cell lines. The uptake of ^124^I-p2B3-Db8 and ^124^I-bm2B3-Db8 in PSCA-positive tumors was lower than that of ^124^I-2B3 minibody in the same tumor model. PET imaging with ^124^I-bm2B3-Db8 visualized the LAPC-9 tumor as early as 4 h post injection with a higher contrast at 12 h post injection [248]. Subsequent affinity maturation of the 2B3 minibody created the A11 anti-PSCA minibody, which showed improved immunoPET performance [249].

^124^I- and ^89^Zr-labeled anti-PSCA A11 minibodies (scFv-CH3 dimer, 80 kDa) were developed and evaluated for quantitative immunoPET imaging of prostate cancer in 22Rv1-PSCA or LAPC-9 xenograft bearing mice. The non-residualizing ^124^I-labeled minibody had lower tumor uptake (3.62 ± 1.18% ID/g 22Rv1-PSCA, 3.63 ± 0.59% ID/g LAPC-9) than the residualizing ^89^Zr-labeled minibody (7.87 ± 0.52% ID/g 22Rv1-PSCA, 9.33 ± 0.87% ID/g LAPC-9. However, the ^124^I-labeled minibody achieved higher imaging contrast because of lower nonspecific uptake and better tumor-to-soft-tissue ratios [250]. In another study, ^124^I-labeled A11 minibody immunoPET imaging was compared with ^18^F-Fluoride bone scans for detecting prostate cancer bone tumors in osteoblastic, PSCA-expressing, and LAPC-9 intratibial xenografts. The ^124^I-labeled A11 minibody demonstrated superior sensitivity and specificity over the ^18^F-Fluoride bone scans in detecting the xenografts at all-time points [251].

A11 cMb was conjugated with the near-infrared fluorescence (NIRF) dye Cy5.5 and radiolabeled with ^124^I or ^89^Zr for evaluation as an immunoPET/fluorescence imaging agent to improve intraoperative prostate cancer margin visualization. ImmunoPET imaging using dual-labeled ^124^I-A11 cMb-Cy5.5 showed specific targeting to both 22Rv1-PSCA and PC3-PSCA. xenografts in nude mice. Similarly, fluorescence imaging showed a strong signal from both 22Rv1-PSCA and PC3-PSCA tumors compared with non-PSCA expressing tumors [252]. Another dual probe, A2 cys-diabody (A2cDb)-IR800, targeting PSCA was labeled with ^124^I (^124^I-A2cDb-IR800) and evaluated in a prostate cancer xenograft model. Dual-modality imaging using the anti-PSCA cys-diabody resulted in high-contrast immuno-PET/NIRF images [253].

### 5.9. Other Biomarkers

#### 5.9.1. Extra Domain-B (ED-B) of Fibronectin

The extracellular matrix protein fibronectin contains a domain, the extra domain B (ED-B) of fibronectin (~80 kDa molecular weight), that is rarely found in healthy adults and is almost exclusively expressed by newly formed blood vessels in tumors, i.e., angiogenesis and different types of lymphoma and leukemias.

The human mAb fragment L19-SIP ((Radretumab) is directed against extra domain B (ED-B) of fibronectin. ^124^I-L19-SIP immunoPET was used to demonstrate its suitability for imaging of angiogenesis at early-stage tumor development and as a scouting procedure before clinical ^131^I-L19-SIP radioimmunotherapy. Tumor uptake, in FaDu xenograft-bearing nude mice, was 7.3 ± 2.1, 10.8 ± 1.5, 7.8 ± 1.4, 5.3 ± 0.6, and 3.1 ± 0.4% ID/g at 3, 6, 24, 48, and 72 h post injection [254]. ImmunoPET imaging with ^124^I-labeled L19SIP was used to predict doses delivered to tumor lesions and healthy organs by subsequent Radretumab RIT in patients with brain metastases from solid cancer. Although the fraction of injected activity in normal organs was similar in different patients, the antibody uptake in the neoplastic lesions varied by as much as a factor of 60 [255].

#### 5.9.2. Phosphatidylserine

Phosphatidylserine (PS) is a marker normally absent that becomes exposed on tumor cells and tumor vasculature in response to oxidative stress in cancer cells (lung, breast, pancreatic, bladder, skin, brain metastasis, rectal adenocarcinoma, etc.) but not on the normal cells. ^124^I-labeled PGN650, an F(ab′)_2_ antibody fragment, was evaluated as a biomarker of the tumor microenvironment. Pharmacokinetics, tumor uptake, and radiation dosimetry in cancer patients were assessed. Apart from the tumor, the liver was found to receive a high radiation dose [256]. Annexin-V, a calcium-dependent protein that binds with high specificity to phosphatidylserine exposed during apoptosis, was labeled with ^124^I for use as a potential PET probe. The biological activity of radiolabeled Annexin-V was tested in control and camptothecin-treated (i.e., apoptotic) human leukemic HL60 cells. A significantly high binding (21%) was observed [257].

#### 5.9.3. Placental Alkaline Phosphatase (PLAP)

Placental alkaline phosphatase (PLAP), also known as an allosteric enzyme that in humans is encoded by the ALPP gene. PLAP is a tumor marker, especially in seminoma and ovarian cancer (e.g., dysgerminoma). The ^124^I-labeled murine mAb H17E2, detecting placental alkaline phosphatase (PLAP), was administered by intraperitoneal injection into nude mice bearing subcutaneous HEp2 human tumor xenografts (a PLAP expressing cell-line). Activity in tumor rose to 4.26% injected dose by 48 h post injection and remained at this level until day 7 post injection, giving a tumor: blood ratio of 0.78 at this time [258].

## 6. Overview of ImmunoPET Imaging Pharmaceuticals for Cancer–Clinical

### 6.1. Receptor Tyrosine Kinase

#### Trastuzumab

^124^I-labeled trastuzumab was evaluated in animals and humans for its application as a potential PET tracer [259]. MicroPET imaging and biodistribution of ^124^I-trastuzumab were performed to examine its specificity in HER2-positive and negative mouse models. Higher tumor uptake of ^124^I-trastuzumab than ^124^I-IgG1 in HER2-positive PDX mouse models at 24 h was seen. The low tumor uptake of ^124^I-trastuzumab in HER2-negative PDX models further confirmed the specificity. ^124^I-trastuzumab was evaluated for its distribution, internal dosimetry, and initial PET imaging of HER2-positive lesions in gastric cancer (GC) patients. PET/CT images of six gastric cancer patients with metastases were compared using ^124^I-trastuzumab and [^18^F]FDG PET/CT. 18 HER2-positive lesions and 11 HER2-negative lesions were evaluated in PET imaging analysis. The detection sensitivity of ^124^I-trastuzumab was 100% (18/18) at 24 h post injection. The PET images showed a significant difference in tumor uptake between HER2-positive and HER2-negative lesions at 24 h post injection. Higher specificity of ^124^I-trastuzumab than [^18^F]FDG was observed.

### 6.2. Glycoproteins

#### Glycoproteins A33 (huA33)

In a clinical study, ^124^I-labeled hu A33 was injected intravenously to 15 patients with colorectal cancer to examine the quantitative features of antibody–antigen interactions in tumors and normal tissues. PET/CT studies showed significant antibody targeting in tumors and normal bowel. There was a linear correlation between the amount of bound antibody and antigen concentration [260]. Targeting, biodistribution, and safety of ^124^I-labeled huA33 in patients with colorectal cancer were evaluated using quantitative PET. Additionally, biodistribution was also determined when a large dose of human intravenous IgG (IVIG) was administered to manipulate the Fc receptor or when ^124^I-labeled huA33 was given via hepatic arterial infusion (HAI). Ten of 12 primary tumors in 11 patients (0.016% ID/g in tumors vs. 0.004% ID/g in normal tissues) were visualized. The HAI route had no advantage over the intravenous route [261]. A novel, nonlinear compartmental model using PET-derived data from 11 patients was developed. The objective of the study was to determine the “best-fit” parameters and model-derived quantities for optimizing biodistribution of intravenously injected ^124^I-labeled A33. Excellent agreement between fitted and measured parameters of tumor uptake was observed [262]. Red marrow activity concentration and the self-dose component of absorbed radiation dose to red marrow were estimated based on PET/CT results of ^124^I-labeled cG250 and huA33. The red marrow-to-plasma activity concentration (RMPR) values were found to be patient-dependent and increase over a 7-day timescale for both the antibodies, indicating that individualized image-based dosimetry is required for optimal therapeutic delivery of radiolabeled antibodies [263].

### 6.3. Carbonic Anhydrase IX

#### cG250

^124^I-labeled cG250 (Girentuximab) was investigated for PET assessment to predict clear-cell renal carcinoma in cancer patients. Twenty-six patients with renal masses who were scheduled to undergo surgical resection were given a single intravenous infusion of ^124^I-cG250. 15 of 16 clear-cell carcinomas were identified accurately by antibody PET. The sensitivity of ^124^I-cG250 PET for clear-cell kidney carcinoma in this trial was 94% [264,265]. Additional clinical studies involving 195 patients validated the safety and superior diagnostic value of ^124^I-cG250 in ccRCC with an average sensitivity and specificity of 86.2% and 85.9%, respectively [266].

Multimodal imaging technique development study, using ^124^I-cG250, concluded that it could realize precise intraoperative localization of ccRCC. This could be clinically very useful to urologic surgeons, urologic medical oncologists, nuclear medicine physicians, radiologists, and pathologists in further guiding and confirming complete evaluation and surgical resection of the diseases [267,268,269]. Furthermore, cG250 (Girentuximab) has been labeled with an assortment of radionuclides (^124^I, ^111^In, ^89^Zr, ^131^I, ^90^Y, and ^177^Lu) and is the most extensively investigated as CA-IX theranostics pharmaceuticals [270].

### 6.4. Other Biomarkers

#### Glypican 3

Glypican-3, a cell-surface glycoprotein in which heparan sulfate glycosaminoglycan chains are covalently linked to a protein core, is overexpressed in hepatocellular carcinoma (HCC) tissues but not in the healthy adult liver. Thus, Glypican-3 is becoming a promising candidate for liver cancer diagnosis and immunotherapy. In a clinical study, ^124^I-codrituzumab (aka GC33), an antibody directed at Glypican 3, was evaluated in 14 patients with hepatocellular carcinoma (HCC). ^124^I-codrituzumab detected tumor localization in most patients with HCC. Pharmacokinetics was similar to that of other intact iodinated humanized IgG [271].

## 7. Summary

In this report, a comprehensive review of the physical properties of iodine and iodine radionuclide, production processes (target selection, preparation, irradiation, and processing), various ^124^I-labeling methodologies for radiolabeling of large biomolecules, (mAbs, proteins, and protein fragments), and the development of immunoPET imaging pharmaceuticals for various cancer targets in preclinical and clinical environments is provided. Several production processes, including ^123^Te(d,n)^124^I, ^124^Te(d,2n)^124^I, ^121^Sb(α,n)^124^I, ^123^Sb(α,3n)^124^I, ^123^Sb(^3^He,2n)^124^I, ^nat^Sb(α,xn)^124^I, ^nat^Sb(^3^He,n)^124^I reactions, have been used in the past. However, as a result of the less frequent availability of deuteron, alpha, and ^3^He beams, ^124^I is being produced, using ^124^Te(p,n)^124^I reaction, successfully for research and clinical use by low-energy cyclotrons. A fully-automated process for the production of ^124^I which can be scaled up for GMP production of large quantities of ^124^I was developed recently. Direct, using inorganic and organic oxidizing agents and enzyme catalysis, and indirect, using prosthetic groups, ^124^I-labeling techniques have been developed and optimized in the past. The Iodogen method is used routinely in research and clinical environments. Significant research has been conducted over more than two decades in the development of immunoPET imaging pharmaceuticals for target-specific cancer detection. ^124^I-labeled Trastuzumab, huA33, and cG250 have shown promise in human clinical trials. There is no FDA approved ^124^I-labeled immunoPET imaging pharmaceutical available. It may be due to (1) availability of manual, difficult, and costly production and purification processes for I-124 in the past, (2) low resolution of PET images due to the high energy of available positrons from I-124, and (3) dehalogenation of ^124^I-labeled mAbs. These bottlenecks have been resolved now by (1) development of a fully-automated process for I-124 production which can be scaled up for the cost-effective GMP production, (2) optimization of image acquisition parameters and appropriate corrections within the image reconstruction process to improve the image quality, and (3) using non internalizing mAbs for development target-specific immunoPET imaging pharmaceuticals. Further future studies in the improvement of safety and efficacy of immunoPET imaging pharmaceuticals and establishment of GMP-compliant I-124 production facilities may bring FDA-approved ^124^I-labeled immnoPET imaging pharmaceuticals to the human clinic use in the future.

## Figures and Tables

**Table 1:** Physical properties and production methods for some cyclotron produced non- metallic and metallic positron (β^+^) emitting radionuclides [2,3,4,5].

Radionuclide	Production Method	Half-Life	%Decay Mode	E_max_ (β^+^), MeV	E_mean_ (β^+^), MeV	Reference
^15^O	^15^N(p,n)^15^O	2.0 min	β^+^/99.9 EC/0.1	1.732	0.725	[2,3]
^13^N	^13^C(p,n)^13^N ^16^O(p,α)^13^N	10.0 min	β^+^/99.8 EC/0.2	1.199	0.492	[2,3]
^11^C	^14^N(p,α)^11^C	20.4 min	β^+^/99.8 EC/0.2	0.960	0.386	[2,3]
^18^F	^18^O(p,n)^18^F	110 min	β^+^/96.9 EC/3.1	0.634	0.250	[2,3]
^124^I	^124^Te(p,n)^124^I	4.2 d	β^+^/22.7 EC/77.3	2.138	0.975	[2,3]
^64^Cu	^64^Ni(p,n)^64^Cu	12.7 h	β^+^/17.5 EC/43.5	0.653	0.278	[2,3,4]
^68^Ga	^68^Zn(p,n)^68^Ga ^68^Ge/^68^Ga Generators	67.8 min	β^+^/88.9 EC/11.1	1.899	0.836	[2,3]
^44^Sc	^44^Ca(p,n)^44^Sc	3.97 h	β^+^/94 EC/6	1.474	0.632	[5]
^66^Ga	^66^Zn(p,n)^66^Ga	9.5 h	β^+^/56.5 EC/43.5	4.15	1.75	[4]
^86^Y	^86^Sr(p,n)^86^Y	14.7 h	β^+^/31.9 EC/68.1	1.221	0.535	[3,4]
^55^Co	^58^Ni(p,α)^55^Co	17.5 h	β^+^/76 EC/24	1.498	0.570	[4]
^89^Zr	^89^Y(p,n)^89^Zr	78.4 h	β^+^/22.7 EC/76.2	0.902	0.396	[2,3,4]
^52^Mn	^52^Cr(p,n)^52^Mn ^nat^Cr(p,x)^52^Mn	5.59 d	β^+^/29.4 EC/77	0.575	0.242	[4]

**Table 2:** A summary of the main emissions of ^124^I (taken from reference [42]).

Decay Type	Energy, keV	Probability
β^+^ 1	1535	12
β^+^ 2	2138	11
γ 1	603	63
γ 2	723	10
γ 3	1510	3
γ 4	1691	11
X	27.2	17
X	27.5	31
ε	866	11
ε	2557	25
ε	3160	24

**Table 3:** Summary of ^124^I production reactions, yield, and impurity profile.

Nuclear Reaction	Effective Energy	Target Material	Enrichment, %	Yield MBq/µAh	Radionuclidic Impurities, %	Reference
^nat^Sb(α,xn)^124^I	22→13	Sb	Nat	0.45 at 5 d EOB	^123^I (4), ^125^I(27), ^126^I(27) at 5 d EOB	[54]
^121^Sb(α,n)^124^I	22→13	Sb	99.45	0.92 at 5 d EOB	^123^I (<4),^125^I(<0.2), ^126^I(<0.2) at 5 d EOB	[54]
^nat^Sb(^3^He,xn)^124^I	35→13	Sb	Nat	0.42 at 5 d EOB	^123^I(14), ^125^I(1.3), ^126^I(1.6)	[55,60]
^123^Sb(α,3n)^124^I	42→32	Sb	98.28	11.7 at EOB	^123^I (<5), ^125^I(<1.8), ^126^I(<0.6) at 60h EOB	[59]
^123^Sb(^3^He,2n)^124^I	45→32	Sb		15.5	^123^I (14), ^125^I(<1.19)	[62]
^123^Te(d,n)^124^I	11→6	Te	91.0, 85.4	2.8 *	^123^I(3321) **	[53]
^124^Te(d,2n)^124^I	15→0	Te	95	0.55	^126^I(0.5)	[44]
	15→8	Te	91.7	18.9	^125^I(0.35) **, ^126^I(0.39) **, ^131^I(0.08) **	[45]
	16→6	Te	96.7	0.64 *	------	[46]
	16–6	TeO_2_	89.6	-	Total Impurities < 5, At 40 h EOB	[49]
	14→0	TeO_2_	89.6	15	^125^I(1.41), ^126^I(1.16), ^130^I (7.87), ^131^I(0.31)	[47]
	13.5→10	TeO_2_	96	-	^125^I(2)	[50]
	14→10	Te	99.8	17.5 *	^125^I(1.7) *	[51]
^124^Te(p,n)^124^I						
	13→9	Te	99.51	20 *	^123^I(41)	[64]
	12.2→0	TeO_2_	99.8	13	^123^I(10.039), ^125^I(0.018), ^126^I(0.041), ^130^I(0.379)	[47]
	14.7	TeO_2_/6.7%A_l2_O_3_	96.7–99.9	20	^123^I(30), ^125^I(<0.1), ^126^I(<0.1), ^130^I(<0.1) ^131^I(<0.1)	[74]
	12.3→9.8	TeO_2_	98	12.5 at 2 d EOB	^123^I(0.5), ^125^I(<0.07) At 2 d EOB	[76]
	13.5→9	TeO_2_/5%Al_2_O_3_	99.8	5.8	^125^I(0.01), ^126^I(<0.0001)	[77]
	12.5→5	TeO_2_	99.8	9.0 ± 1.0	^125^I(0.053)	[78]
	14→7	TeO_2_/5%Al_2_O_3_	99.86	21.1	^125^I(0.03), ^126^I(0.007)	[80]
	11→2.5	TeO_2_/6%Al_2_O_3_	99.5	6.40 ± 0.44	^125^I(<0.02), ^126^I(<0.001)	[81]
	11→2.5	Al_2_Te_3_	99.5	8.47	^125^I(<0.001), ^126^I(<0.001)	[82]
	11.6→0	TeO_2_/5%Al_2_O_3_	99.8	6.88	^123^I	[84]
	12.6	TeO_2_	99.8	13.0	^123^I (0.18), ^125^I (0.037) ^126^I (0.0099)	[85]
	16.5→12	TeO_2_/5%Al_2_O_3_	99.93	4–5	^123^I (<1.5), ^125^I (<0.001)	[86]
^125^Te(p,2n)^124^I	20.1→10.5	TeO_2_	93	43.3	^123^I(8), ^125^I(5)	[87]
	22→4	Te	98.3	111 *	^125^I(0.89)	[89]
	21→15	Te	98.3	81 *	^123^I(7.4), ^125^I(0.9)	[65]
	22	TeO_2_	98.5	104	^123^I(<1)	[88]
^126^Te(p,3n)^124^I						
	38→28	Te	98.69–99.8	222 *	^123^I(148), ^125^I(1.0), ^126^I(1.0)	[61]
	36.8→33.6	Te	nat	67 *	--	[91]

* Based on cross-section data, ** Percent calculated here from the ratio of the published saturation yield data.

## Data Availability

No new data were created or analyzed in this study. Data sharing is not applicable to this article.
